# Immobilized horseradish peroxidase hybrid biocatalysts for degradation of pharmaceuticals and other emerging micropollutants

**DOI:** 10.1371/journal.pone.0355285

**Published:** 2026-09-02

**Authors:** Khadega A. Al-Maqdi, Rana Morsi, Hao Zhou, Assina Abdussaitova, Iltaf Shah, Syed Salman Ashraf

**Affiliations:** 1 Department of Chemistry, College of Sciences, UAE University, Al Ain, United Arab Emirates; 2 Department of Biological Sciences, College of Medicine and Health Sciences, Khalifa University, Abu Dhabi, United Arab Emirates; 3 Biotechnology Center (BTC), Khalifa University, Abu Dhabi, United Arab Emirates; Institute of Biophysics Chinese Academy of Sciences, CHINA

## Abstract

With the increasing detection of emerging pollutants (EPs), such as pharmaceuticals, personal care products, and pesticides, in water bodies, there is a growing research priority to develop efficient methods for their degradation. In the present study, the degradation of 21 EPs from different classes was investigated using horseradish peroxidase (HRP) covalently immobilized onto two photocatalytic supports, TiO_2_ and ZnO. The hybrid biocatalysts (TiO_2_-HRP and ZnO-HRP) were used to degrade EPs with and without the presence of a redox mediator, 1-hydroxybenzotriazole (HOBT). The results revealed that not all EPs were equally degraded by HRP enzymes, with some pollutants showing complete, partial, or no degradation. For example, full degradation was achieved for MBT (100%), meloxicam (99.5%) and caffeic acid (98.6%). In contrast, prometryn and MCPA showed only partial degradation, reaching 19.2% and 20%, respectively, and no degradation was observed for fluometuron and atenolol. The addition of the model redox mediator, HOBT, improved the degradation of some pollutants, for example, lincomycin-HCl degradation increased to 36.9%, while trimethoprim degradation improved to 21.5%. Additionally, several pollutants, including roxithromycin, cimetidine, and caffeine, exhibited significantly higher degradation rates when treated with the immobilized HRP enzyme (TiO_2_-HRP or ZnO-HRP) compared to free HRP. For instance, roxithromycin degradation increased from 0.5% to 58.2% with ZnO-HRP, and for cimetidine, TiO₂-HRP improved degradation from 0.3% to 60.1% without HOBT and from 9.0% to 66.7% with HOBT. Molecular docking with the catalytic heme cofactor retained further suggested that degradation trends were not explained by docking score alone, but were influenced by ligand access to the distal heme pocket, molecular planarity, and contact topology near catalytic residues. Finally, an integrated HRP enzyme and chemical oxidation remediation strategy was applied to degrade two pollutants, trimethoprim and DEET. For trimethoprim, TiO_2_-HRP combined with H_2_O_2_ and UV achieved approximately 30% degradation, while for DEET, both TiO_2_-HRP + H_2_O_2_ + UV and ZnO-HRP + H_2_O_2_ + UV resulted in around 40% degradation, thereby highlighting a novel application for these hybrid peroxidase-photocatalysts.

## Introduction

Emerging pollutants (EPs) are a group of organic contaminants frequently detected in water bodies worldwide. These pollutants encompass a wide range of organic compounds and their byproducts, including pesticides, pharmaceuticals, hormones, textile dyes, personal care products, along with other possibly toxic substances [1,2]. Numerous studies have highlighted the dangers these pollutants pose to both human and aquatic life, as they can disrupt the endocrine system, cause reproductive issues, lead to physical deformities, and even feminize certain fish species [3]. The concentrations of these pollutants in water bodies vary from ng/L to several hundred µg/L [4]. For example, the antibiotic sulfadiazine has been found in concentrations ranging from 19.5 to 187.0 ng/L [5], while non-steroidal anti-inflammatory drugs like ibuprofen and naproxen have been detected in the ranges of 4.27 to 510 ng/L and 3.19 to 2000 ng/L, respectively [6]. In addition, various pesticides have been identified in Lake Vistonis, including fluometuron (0.088 µg/L), lindane (0.030 µg/L), and lambda-cyhalothrin (0.041 µg/L) [7].

Various physical and chemical methods have been used for the degradation of emerging pollutants in water. However, these methods have limitations, including high energy and operational costs, as well as the generation of large amounts of sludge. This highlights the need for the development of greener, more efficient, and environmentally friendly methods for wastewater treatment, a priority for industrial chemists and researchers [8]. One promising area of research is the use of biological approaches, particularly oxidoreductase enzymes, which have shown potential in degrading a wide range of emerging pollutants. In recent years, several enzymes have been utilized for the effective breakdown of various pollutants into smaller, less harmful intermediates. The use of enzymes in bioremediation offers several advantages, including lower energy consumption, reduced sludge production, and decreased toxicity [9].

Oxidoreductases such as horseradish peroxidase (HRP), soybean peroxidase (SBP), manganese peroxidase (MnP) and laccase have been studied for the degradation and detoxification of a range of emerging pollutants [10,11]. For example, Zhou et al. reported the complete degradation (100%) of doxycycline and tetracycline (antibiotics) by MnP and laccase enzymes [12]. In another study, the laccase enzyme was able to degrade 98% of ketoconazole (an antifungal) [13]. Horseradish peroxidase has been extensively studied for its capability to degrade a wide range of emerging pollutants. Numerous studies have reported high degradation efficiencies, including 100% for catechol, 56.3% for 2,4-dichlorophenol, 44.6% for 4-methoxyphenol, 15.3% for bisphenol A [14], 90% for 17α-ethinylestradiol [15], and 97% for triclosan [16]. Moreover, HRP has been shown to achieve 100% degradation of Estrone, 17β-estradiol, estriol, and synthetic 17α-ethinylestradiol [17]. Additionally, multiple studies have shown a reduction in the toxicity of emerging pollutants when treated with oxidoreductase enzymes [18]. Although lignin peroxidase (LiP) has a higher oxidation potential compared with HRP and may be more effective for the degradation of some EPs [19], HRP was selected in this study because it is a well-characterized, commercially available heme peroxidase with established use in pollutant degradation. Nevertheless, using enzymes for water remediation presents challenges, including issues with enzyme reusability, potential conformational changes, and loss of enzyme activity due to harsh environmental conditions such as extreme pH values, high temperatures, and high ionic strength. Some of these challenges can be overcome by immobilizing enzymes onto solid supports. In enzyme immobilization, the enzyme is attached to an inert, insoluble support material, which restricts or completely eliminates enzyme mobility. This approach has several benefits, including enzyme reusability, the ability to recover enzymes, and improved resistance to harsh pH and temperature conditions. For example, immobilization of HRP on alginate–cobalt ferrite nanocomposites significantly enhanced its stability, reusability, and solvent tolerance compared to the free enzyme, retaining 71% activity after 10 cycles, 74% activity after 8 weeks of storage at 4 °C, and 149% activity in n-hexane [20]. These improvements highlight how enzyme immobilization can enhance enzymatic activity, making enzymes more suitable for large-scale applications [21,22].

The present work was conducted to enhance the potential applications of peroxidase enzymes by immobilizing HRP onto two photocatalytic supports (TiO_2_ and ZnO) to overcome the challenges concerning scalability and the limited reusability of enzyme-based methods. The first aim of this work is to fully characterize the synthesized hybrid biocatalysts (TiO_2_-HRP and ZnO-HRP) regarding their surface morphology, crystallinity, pH, and temperature stability. The second aim is to degrade a mixture of 21 EPs using the immobilized biocatalysts, with and without the presence of a redox mediator, specifically, 1-hydroxybenzotriazole (HOBT). The 21 EPs were selected to mimic the structural diversity and chemical recalcitrance of EPs commonly found in real wastewater streams. The third aim is to compare the degradation efficiency of free HRP enzyme with that of HRP immobilized on TiO_2_ and ZnO. The final objective is to investigate the degradation of EPs through photocatalytic oxidation using UV light and to compare this with the performance of the immobilized HRP. Importantly, this study demonstrates for the first time that immobilizing HRP on TiO_2_ and ZnO photocatalytic supports can markedly enhance the degradation of previously recalcitrant pollutants. Furthermore, by applying this hybrid peroxidase-photocatalyst system to a panel of 21 structurally diverse emerging pollutants, this work provides one of the most comprehensive evaluations of enzyme-photocatalyst synergy for water remediation.

## Materials and methods

### Reagents

All EPs, glutaraldehyde, and (3-aminopropyl)triethoxysilane (APTES) were purchased from Sigma-Aldrich (Fremont, CA, USA). The 21 EPs were purchased standards used to prepare a model pollutant mixture, not compounds collected or quantified from wastewater samples. High-purity solvents for LC-MS, including acetonitrile, water, and formic acid were obtained from Sigma-Aldrich, along with hydrogen peroxide (30% w/v). The photocatalysts used (TiO_2_ and ZnO) were also bought from Sigma-Aldrich. All experiments used universal buffers containing 0.2 M K_2_HPO_4_ and 0.1 M citric acid.

Detailed supplier information, catalog numbers, CAS numbers, purity/grade, and stock-solution preparation details for the 21 EP standards are provided in S1 Table.

### Immobilization of HRP onto photocatalytic supports (TiO_2_ and ZnO)

**Fig 1:**
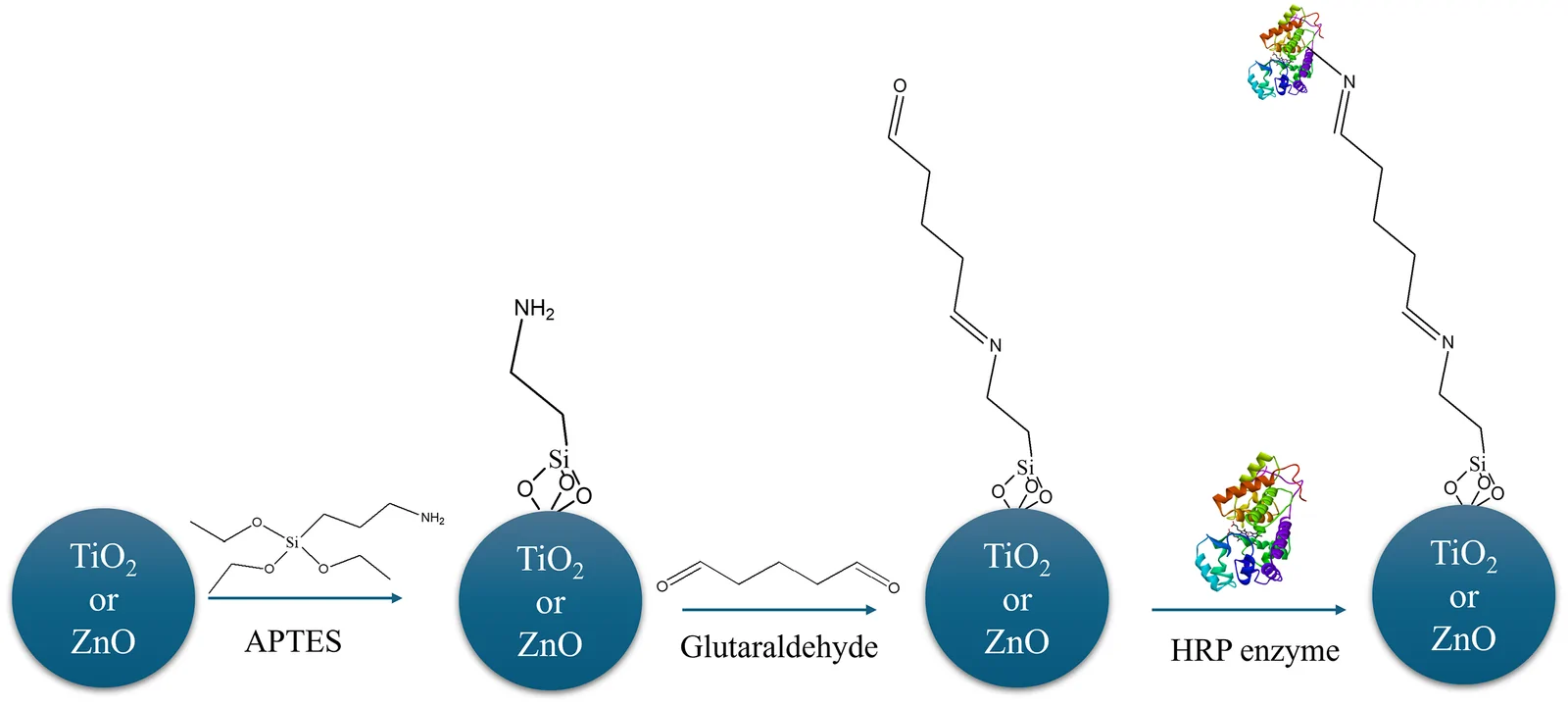
Preparation overview of immobilized hybrid biocatalysts (TiO_2_-HRP and ZnO-HRP).

HRP enzyme was attached to the surface of functionalized photocatalytic supports TiO_2_ and ZnO, as illustrated in Fig 1, based on the method established by Jorge et al. [23]. In brief, 3.0 g of commercial-grade TiO_2_ and ZnO were combined in a 1:1 ethanol/water solution. The solution was then exposed to nitrogen and sonicated for a couple of minutes. Subsequently, 11.12 mL of APTES was incorporated to the mixture, which was left under stirring for 2 hours at 40°C. Next, 1.0 g of the fabricated TiO_2_-APTES and ZnO-APTES was introduced into a 0.1 L solution of glutaraldehyde in 0.1 M phosphate buffer (pH 7) and stirred in the dark for 1 hour, followed by filtration using a Buchner funnel. The resulting solids were added to 40 mL of HRP enzyme solution in phosphate buffer and stirred overnight. Afterward, the precipitated solids were washed three times with phosphate buffer to remove any unbound HRP enzyme. Finally, TiO_2_-HRP and ZnO-HRP were collected and stored at 4°C for further analysis.

### Characterization of photocatalytic supports (TiO_2_ and ZnO) and hybrid biocatalysts (TiO_2_-HRP and ZnO-HRP)

The surface morphology of three materials was analyzed using scanning electron microscopy (SEM): (1) photocatalytic supports (TiO_2_ and ZnO), (2) functionalized photocatalysts (TiO_2_-APTES and ZnO-APTES), and (3) hybrid biocatalysts (TiO_2_-HRP and ZnO-HRP). For SEM analysis, the samples were mounted onto a sample holder using carbon tape and afterward coated with a thin layer of gold. Surface morphology images were obtained with an FEI SEM Quanta Inspect S50 instrument at a voltage of 25 kV. The crystalline structure of all samples was examined using a Shimadzu-6100 X-ray powder diffractometer with Cu-Kα radiation, scanning across a 2θ range of 10° to 80°. Additionally, functionalization of the photocatalytic supports and the immobilization of the enzyme was confirmed by FTIR analysis.

### Degradation efficiency of EPs using free and immobilized HRP enzyme

A mixture of 21 emerging pollutants was treated with both free HRP enzyme and immobilized hybrid biocatalysts (TiO_2_-HRP, ZnO-HRP), with and without the presence of a redox mediator, HOBT, to evaluate the degradation capability of both the free and immobilized HRP enzymes. Each experiment was performed in triplicate to ensure reproducibility, and the total reaction volume for each run was 3 mL. Degradation experiments with the free HRP enzyme were conducted as follows: HRP enzyme (0.36 μM) was added to a mixture of 21 EPs (2 ppm), H_2_O_2_ (0.3 mM), and universal buffer (pH 5). In experiments involving the redox mediator, 0.1 mM HOBT was added to the reaction mixture. For degradation experiments with the immobilized hybrid biocatalysts (TiO_2_-HRP, ZnO-HRP), the procedure was the same as for the free HRP enzyme, except that 20 mg of the hybrid biocatalyst was added in place of the liquid HRP enzyme. Also, the H_2_O_2_ concentration was increased to 0.6 mM. The universal buffer was set to pH 5 for TiO_2_-HRP, and for ZnO- HRP it was set to pH 4. The reaction mixtures were maintained at 25°C for 30 minutes, after which the samples were filtered and analyzed using LC-MS/MS.

For experiments involving UV-photolytic degradation, 5 mg of TiO_2_, ZnO, TiO_2_-HRP, and ZnO-HRP were added to a mixture of 21 EPs (2 ppm) and H_2_O_2_ (0.3 mM). Samples were exposed to a UV lamp (UVGL-58, J-129, Upland, NJ, USA) positioned 1.5 cm from the samples. The UV lamp had a power output of 6 W, with a selected output mode of 254 nm. Under these conditions, the irradiated samples showed no signs of warming.

Although environmental concentrations of these pollutants are typically in the ng/L to µg/L range, a concentration of 2 ppm was selected in this study to facilitate reliable analytical detection and to clearly evaluate the catalytic degradation performance under controlled laboratory conditions.

### Degradation analysis using LC-MS/MS method

After treating the 21 EPs with free and immobilized HRP enzymes (TiO_2_-HRP and ZnO-HRP), the samples were studied by LC-MS/MS using multiple reaction monitoring (MRM) mode, as detailed previously [24]. Prior to injection into the LC-MS system, all samples were filtered through 0.45 μm cellulose syringe filters. A ZORBAX Eclipse Plus C18 column was used with the following specifications: length of 50 mm, particle size of 1.8 μm and inner diameter of 2.1 mm for separation. The temperature of the column was kept at 35°C, and the mobile phase flow rate was set to 0.4 mL/min. The MS detector utilized was an Agilent 6420 Triple Quadrupole mass detector. The method utilized two mobile phases: mobile phase (A) consisting of LC-MS grade water with 0.1% formic acid, and mobile phase (B) consisting of 100% LC-MS grade acetonitrile. The MRM analysis was programmed as follows: 0–2.5 minutes, 0% (B); 2.5–15 minutes, 0–80% (B); 15–18 minutes, 80–90% (B); 18–20 minutes, 90–5% (B); 20–27 minutes, 0% (B). Both positive and negative ionization modes were employed to analyze the EPs samples. Nitrogen gas was utilized for fragmentation, with a capillary voltage of 4000 V, gas flow at 8 L/min and nebulizer pressure at 45 psi.

### Computational methods

A computational workflow was developed to examine how twenty-one emerging pollutants interact with HRP under both free and immobilized conditions. Chemical structures for all pollutants were curated to ensure consistency with the experimental compounds. Each compound name was standardized, salts and hydrates were removed to yield the neutral parent species, and abbreviations such as MBT and SMX were expanded to full chemical names. Canonical SMILES were verified, and three-dimensional ligand geometries were generated using Open Babel; protonation states were assigned within the pH range 4.0–5.0, corresponding to the mildly acidic conditions of the HRP assays. For each ligand, relevant protonation and tautomeric microstates were enumerated with Dimorphite-DL, and individual PDBQT files were produced for docking. Two crystallographic HRP structures were employed: the resting ferric state (PDB 1ATJ) and the reactive Compound I state (PDB 1HCH). Both were processed with PDBFixer and OpenMM to repair missing atoms and add hydrogens at pH 4.5, followed by conversion to AutoDock-compatible PDBQT format via Meeko. The heme iron atom was used as the reference point for grid construction. Two simplified docking environments were defined for each receptor: a 22 Å cubic box representing the accessible “free” enzyme model, and an 18 Å cubic box representing an “occluded” model used to approximate steric constraints from nanoparticle immobilization.

Each ligand microstate was docked against both HRP structures in both configurations using AutoDock Vina [25] on a high-performance computing cluster. All combinations of receptor, grid, and ligand were automatically enumerated and executed. For every docked complex, the best AutoDock Vina score (kcal mol^-1^) and the minimum heavy-atom distance from the ligand to the heme Fe were extracted. Post-processing scripts characterized the docked ligands by physicochemical and geometric descriptors. RDKit was used to compute molecular weight, polarity (TPSA), rotatable bonds, aromatic ring count, and hydrogen-bond donors/acceptors. Ligand geometry was analyzed to estimate span, molecular volume, and planarity, the latter quantified as the ratio of the smallest to the largest eigenvalue of the atomic coordinate covariance matrix.

Three-dimensional visualization of HRP–ligand interactions was performed using the PyMOL Molecular Graphics System [26]. The crystal structure of HRP (PDB ID: 1ATJ) was obtained from the Protein Data Bank [27]. Protein structures were rendered as cartoons, ligands as sticks, and the heme group as red sticks. Residues within 4 Å of each ligand were defined as contacting residues and were highlighted to identify interaction patterns and conserved binding features within the catalytic pocket.

Secondary structure assignments of contacting residues were categorized into α-helices, β-sheets, coils, and turns (Supplementary Tables S4 and S5). Comparative analyses were conducted between compounds exhibiting high degradation efficiencies (>50%) and those with low degradation efficiencies (<20%). Statistical differences between the groups were evaluated using the Mann–Whitney U test. To quantify structural similarity between ligands and the reference substrate (guaiacol), residue-overlap analysis was performed using the Jaccard similarity index [28].

## Results and discussion

### LC-MS/MS methodology development

Various classes of EPs have been frequently detected in numerous water bodies, including pesticides, pharmaceuticals, personal care products, hormones and many others. These pollutants are considered to pose significant risks to human health and ecological systems [29,30]. In this work, our focus was on the degradation of 21 different emerging pollutants using free HRP enzyme and immobilized HRP enzyme on two photocatalytic supports, namely TiO_2_-HRP and ZnO-HRP. The pharmaceutical classes of the 21 EPs tested are shown in Table 1, while their toxicity and environmental relevance are summarized in S2 Table. To quantify the EPs and evaluate their degradation by free and immobilized HRP enzyme, a sensitive and robust LC-MS/MS method, previously described by [24], was employed. S3 Table provides an overview of the MRM method development for all 21 EPs, providing details on their MRM parameters, such as precursor ion, product ion, polarity, and collision energy. S1 Fig displays the full chromatogram of the 21 EPs mixture, while S2 Fig presents the extracted chromatogram for each pollutant tested.

**Table 1:** Chemical names and uses of the 21 EPs used in the present study.

EPs	Uses	EPs	Uses
Roxithromycin	Antibiotic	Fluometuron	Herbicide
Lincomycin hydrochloride (Lincomycin-HCl)	Antibiotic	Ibuprofen	NSAID
Meloxicam	Nonsteroidal anti-inflammatory drug (NSAID)	Thiabendazole	Fungicide
Norfloxacin	Antibiotic	2-Methyl-4- chlorophenoxyacetic acid (MCPA)	Herbicide
Trimethoprim	Antibiotic	Caffeine	Stimulant
Venlafaxine hydrochloride (Venlafaxine-HCl)	Antidepressant	DEET	Insect repellent
Atenolol	Beta blocker medication	Caffeic acid	Antioxidant
Sulfamethoxazole (SMX)	Antibiotic	Mercaptobenzothia-zole (MBT)	Sulfur vulcanization of rubber
Cimetidine	Histamine H₂ receptor antagonist	Furosemide	Loop diuretic medication
Phenytoin	Anti-seizure medication	Hydrochlorothiazi-de	Diuretic medication
Prometryn	Herbicide		

### HRP-mediated degradation of 21 EPs

As previously stated, numerous research teams have demonstrated that peroxidases and laccases can degrade a wide range of EPs [31–33]. The present study aims to further explore the potential use of peroxidases through the immobilization of HRP onto photocatalytic supports (TiO_2_ and ZnO) to address some of the challenges encountered with enzyme-based approaches.

**Fig 2:**
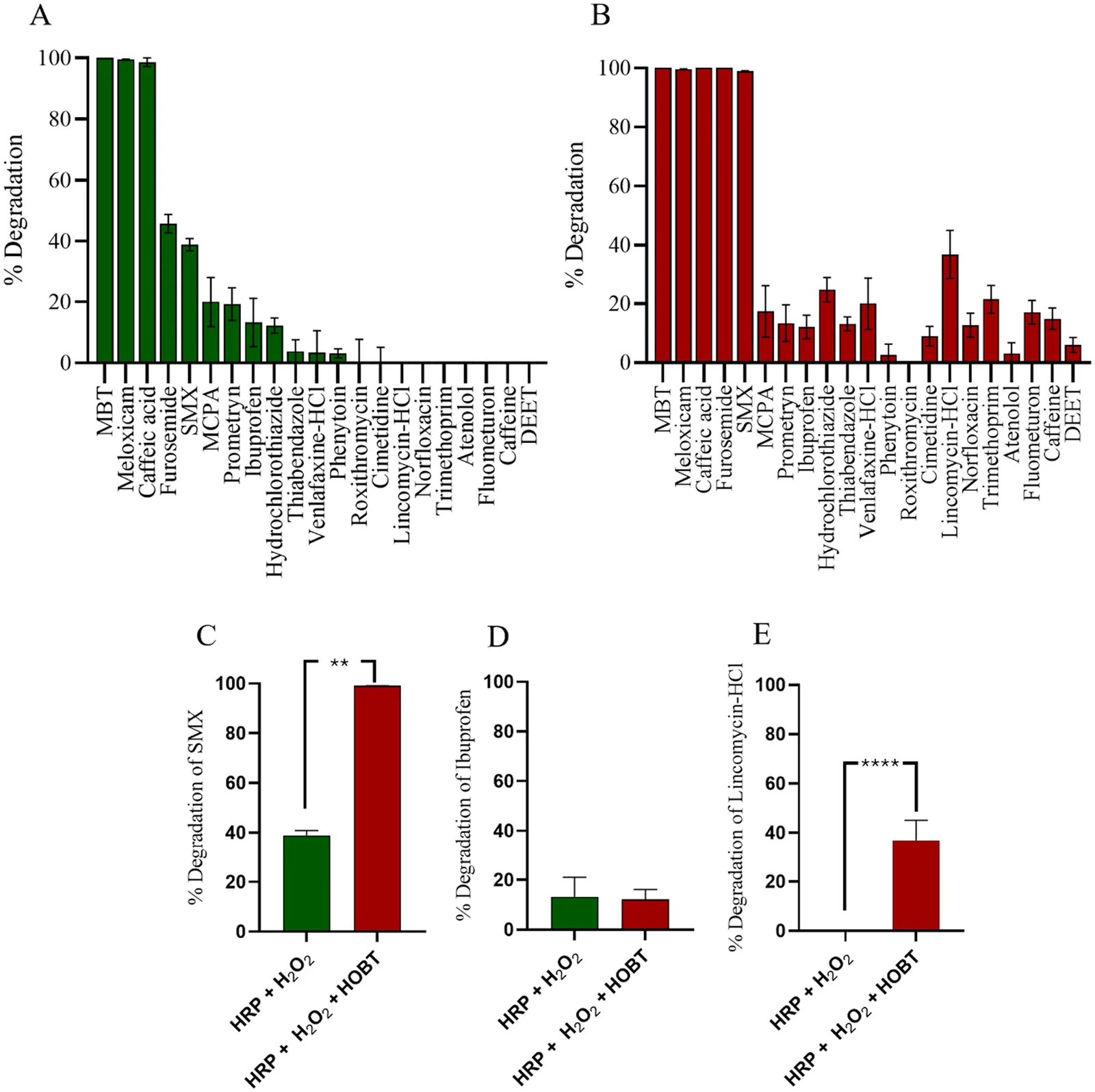
A. The degradation of 21 EPs by HRP alone, B. The degradation of 21 EPs by HRP + HOBT, C. The degradation of SMX with and without HOBT, D. The degradation of ibuprofen with and without HOBT, E. The degradation of lincomycin-HCl with and without HOBT. Each experiment was done in triplicates and the error bars represent ± one standard deviation (SD). The asterisk (*) shows the significant difference, where p < 0.05 (*), p < 0.01 (**), p < 0.001 (***), and p < 0.0001 (****).

A mixture of 21 EPs was treated with HRP to examine its capability to degrade them. HRP enzyme was successful in significantly degrading several EPs (greater than 90%), while others were either moderately degraded (10–90%) or not degraded at all (less than 10%). Fig 2A illustrates the results found when a mixture of the 21 EPs was treated with HRP + H_2_O_2_ for 30 minutes. According to the findings, it is observable that HRP efficiently degraded mercaptobenzothiazole (MBT) (100% ± 0.0), meloxicam (99.5% ± 0.1), and caffeic acid (98.6% ± 1.4). It similarly catalyzed the moderate degradation of furosemide (45.8% ± 3.0), sulfamethoxazole (SMX) (38.9% ± 2.0), and MCPA (20.0% ± 8.1). A number of other pollutants were degraded, but to a much lower amount, such as prometryn (19.2%), ibuprofen (13.2%), and hydrochlorothiazide (12.2%). The remaining 12 EPs demonstrated negligible degradation (less than 10%) with HRP + H_2_O_2_ treatment.

Previous studies have also reported the ability of peroxidase-type enzymes to degrade several emerging pollutants. For example, engineered heme enzymes with peroxidase activity have achieved very high degradation efficiencies for compounds such as 2-mercaptobenzothiazole and furosemide, with degradation rates approaching 99% and 91%, respectively [34]. Similarly, HRP has been reported to effectively degrade mercaptobenzothiazole and caffeic acid (>75%), while moderate degradation has been observed for compounds such as sulfamethoxazole and furosemide in the absence of redox mediators [24]. These findings are consistent with the trends observed in the present study.

Although this approach uses a complex mixture of 21 emerging pollutants to simulate a multi-contaminant system, the findings should be interpreted with caution. For instance, the “lack of significant degradation” observed for the 12 EPs could be the result of inhibition or competition among the different EPs used and may not necessarily indicate HRP's failure to degrade them.

### Requirement of a redox mediator (RM)

Redox mediators are small organic compounds that assist in oxidoreductase-driven reactions. Commonly used redox mediators include HOBT, 2-methoxyphenothiazone, and veratryl alcohol. Numerous investigations have shown that the presence of a redox mediator in peroxidase-based reactions can influence the degradation of EPs in different ways. They may enhance pollutant degradation, inhibit it, or have no effect at all [35–37].

In this study, the role of HOBT in the efficiency of HRP-assisted degradation of 21 selected EPs was investigated. Fig 2A and 2B compare the degradation of these EPs by the HRP enzyme with and without HOBT. For instance, as shown in Fig 2C, the presence of HOBT significantly boosted the degradation rate of SMX. HRP alone degraded ~40% of SMX, but the addition of HOBT increased degradation to 100%. A similar trend was observed for furosemide, where degradation increased from ~45% to 100%. Consistent with previous findings, the addition of HOBT dramatically improved the degradation of many EPs by HRP, lignin peroxidase (LiP), Chloroperoxidase (CPO), and SBP [24]. However, HOBT had no significant impact on ibuprofen degradation, with percentages remaining consistent (~12%) with and without HOBT (Fig 2D). Similarly, no effect was observed for MBT, meloxicam, prometryn, and caffeic acid. For some pollutants, including lincomycin-HCl (Fig 2E), hydrochlorothiazide, trimethoprim, fluometuron, caffeine, thiabendazole, and norfloxacin, HRP alone showed no degradation. However, the addition of HOBT enabled partial degradation (13–37%), which, although modest, offers hope for better results with optimized reaction conditions, such as extended reaction times. Interestingly, the observed improvement with HOBT is statistically significant (p < 0.05 or more significant), indicating that its presence meaningfully enhances HRP-mediated degradation for these otherwise recalcitrant pollutants. Lastly, four pollutants phenytoin, roxithromycin, cimetidine and atenolol exhibited no significant degradation (less than 10%) under the tested conditions. Table 2 shows the statistical comparison between HRP and HRP + HOBT treatments.

**Table 2:** Statistical comparison (t-test) between HRP and HRP + HOBT treatments showing mean ± SD and significance for pollutant degradation efficiency. The asterisk (*) shows the significant difference, where p < 0.05 (*), p < 0.01 (**), p < 0.001 (***), and p < 0.0001 (****). “N.S.” denotes not significant.

Emerging Pollutants	HRP % Degradation	HRP + HOBT % Degradation	Significance (*)
MBT	100 ± 0.0	100 ± 0.0	N.S.
Meloxicam	99.5 ± 0.1	99.6 ± 0.1	N.S.
Caffeic acid	98.6 ± 1.4	100 ± 0.0	N.S.
Furosemide	45.8 ± 3.0	100 ± 0.0	****
SMX	38.9 ± 2.0	99 ± 0.1	****
MCPA	20 ± 8.1	17.4 ± 8.7	N.S.
Prometryn	19.2 ± 5.3	13.4 ± 6.2	N.S.
Ibuprofen	13.2 ± 7.9	12.1 ± 4.0	N.S.
Hydrochlorothiazide	12.2 ± 2.5	24.8 ± 4.2	**
Thiabendazole	3.8 ± 3.7	13.2 ± 2.3	*
Venlafaxine-HCl	3.3 ± 7.2	20.1 ± 8.8	N.S.
Phenytoin	3.1 ± 1.5	2.6 ± 3.7	N.S.
Roxithromycin	0.5 ± 7.2	0.0 ± 0.0	N.S.
Cimetidine	0.3 ± 4.8	9.0 ± 3.3	N.S.
Lincomycin-HCl	0.0 ± 0.0	36.9 ± 8.1	***
Norfloxacin	0.0 ± 0.0	12.7 ± 4.1	**
Trimethoprim	0.0 ± 0.0	21.5 ± 4.7	***
Atenolol	0.0 ± 0.0	3.1 ± 3.7	N.S.
Fluometuron	0.0 ± 0.0	17.1 ± 4.0	***
Caffeine	0.0 ± 0.0	14.9 ± 3.6	***
DEET	0.0 ± 0.0	6.0 ± 2.5	*

### Characterization of hybrid biocatalysts (TiO_2_-HRP and ZnO-HRP)

#### Scanning electron microscope (SEM).

**Fig 3:**
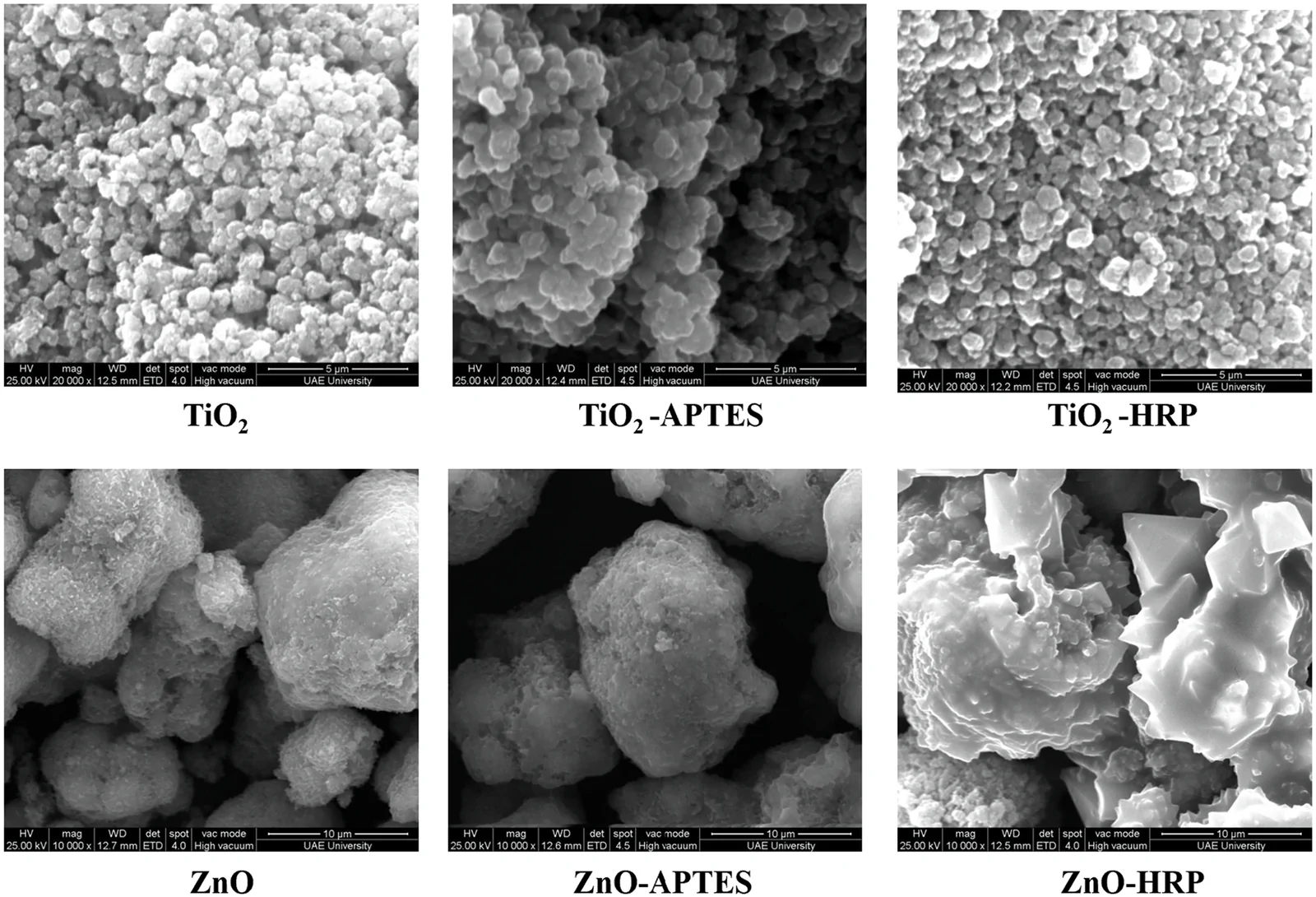
SEM images of the photocatalysts (TiO_2_ and ZnO), functionalized photocatalysts (TiO_2_ APTES and ZnO-APTES) and immobilized HRP enzyme on the photocatalysts (TiO_2_-HRP and ZnO-HRP).

We obtained SEM images to examine the morphology of the photocatalysts, APTES-functionalized photocatalysts, and HRP-hybrid biocatalysts. As shown in the SEM images (Fig 3), TiO_2_ nanoparticles have a uniform, agglomerated, spherical morphology, whereas ZnO nanoparticles are considerably larger and more irregular. The APTES functionalization produced a smoother surfaces and reduced aggregation for photocatalysts. Immobilization of HRP increases surface roughness and aggregation, with TiO_2_-HRP showing minor roughness and ZnO-HRP displaying a more fragmented, flaky structure. Remarkably, the immobilization of HRP on ZnO resulted in a significant change in morphology, unlike TiO_2_.

#### X-ray diffraction (XRD).

**Fig 4:**
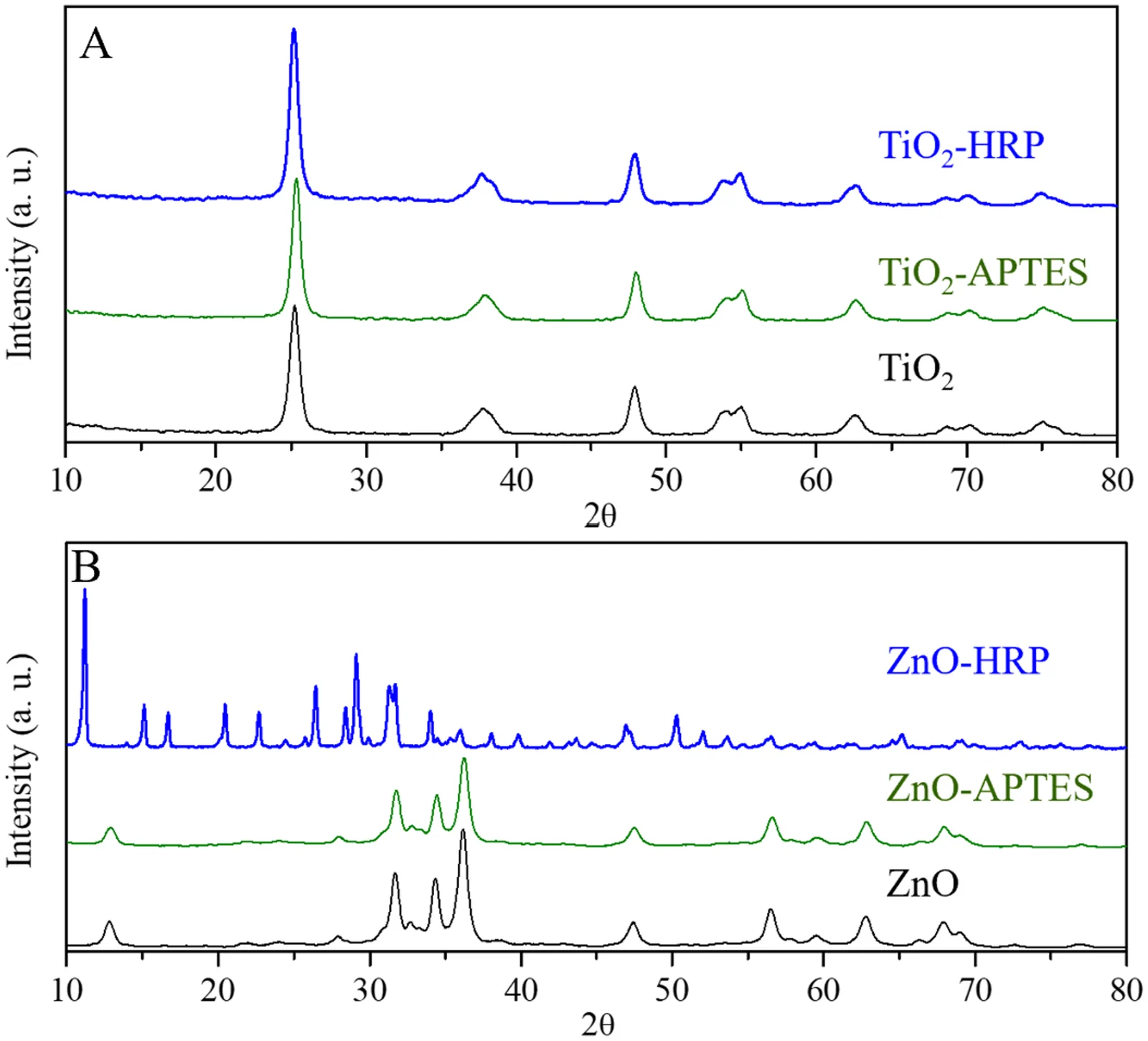
XRD patterns of A. pure TiO_2_, TiO_2_-APTES, and TiO_2_-HRP, B. pure ZnO, ZnO-APTES, and ZnO-HRP.

Fig 4A and B show the XRD patterns of the photocatalysts, APTES-functionalized photocatalysts, and HRP-hybrid biocatalysts. The results indicate that the functionalization of TiO_2_ and ZnO with APTES did not lead to any significant changes in the XRD patterns when matched to the standard XRD patterns for neat TiO_2_ and ZnO [38,39]. When HRP was immobilized on the functionalized photocatalysts using glutaraldehyde, the crystallinity of TiO_2_-HRP remained unchanged. However, the immobilization of the enzyme onto ZnO showed a strong interaction/reaction between ZnO and HRP.

Overall, both the SEM and XRD results correlate to a strong reaction between APTES-functionalized ZnO and ZnO-HRP when glutaraldehyde was used for immobilization, as evidenced by the distinct shapes and crystallinities of ZnO-HRP compared to neat ZnO. The XRD spectrum of the ZnO-HRP sample shows the wurtzite ZnO phase with no new crystalline impurities. Compared with neat ZnO, Bragg intensities are reduced and slightly broadened, attributable to (i) dilution by an amorphous HRP overlayer and (ii) microstructural effects (surface disorder/texture) introduced by immobilization. The minor changes in relative peak intensities between the neat and HRP-immobilized ZnO XRD spectra only reflect packing/texture differences, not a phase transformation, as has also been noted in previous studies [40,41].

#### FTIR.

**Fig 5:**
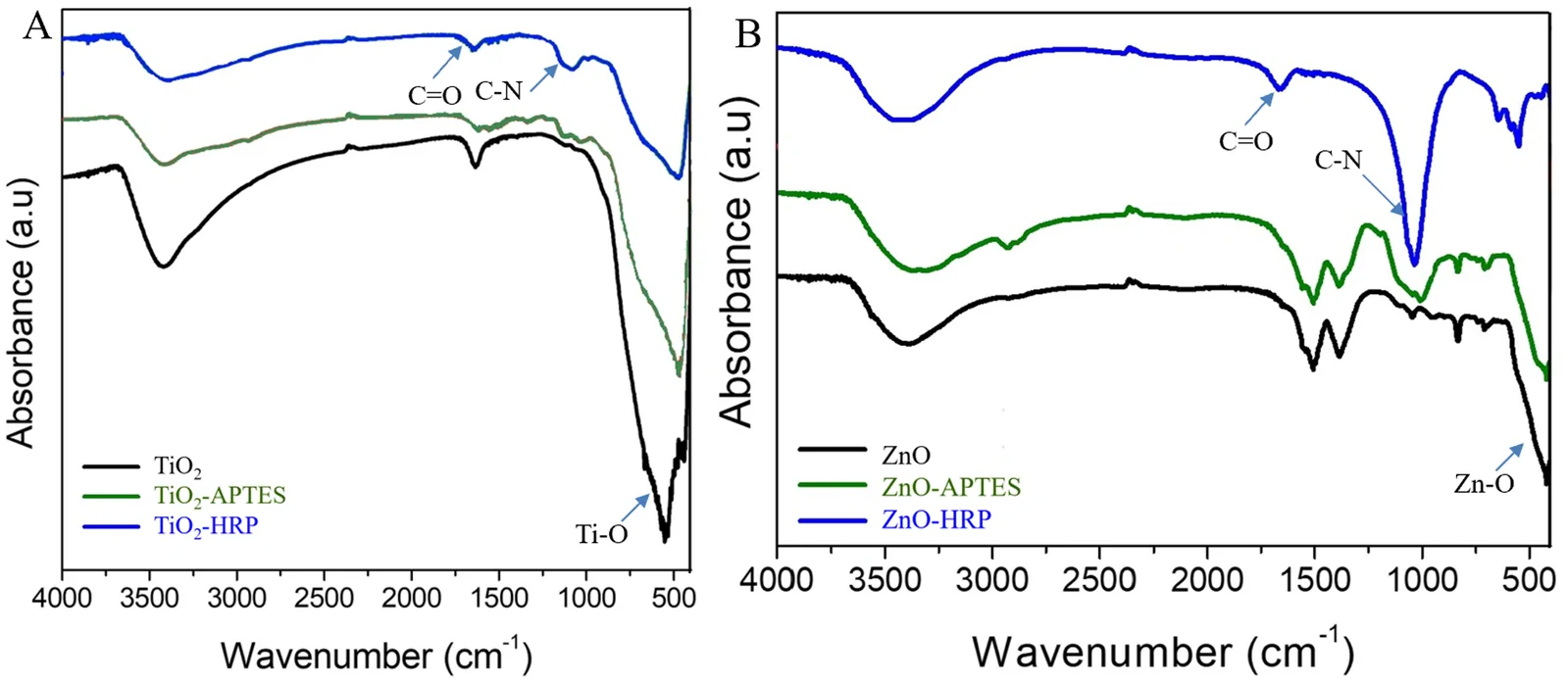
FTIR spectra of A. TiO_2_, TiO_2_-APTES and TiO_2_-HRP, B. ZnO, ZnO-APTES and ZnO-HRP.

The successful immobilization of the HRP enzyme onto the photocatalysts (TiO_2_ and ZnO) was confirmed by comparing the FTIR spectra of the hybrid biocatalysts (TiO_2_-HRP and ZnO-HRP) with those of neat TiO_2_ and ZnO, as shown in Fig 5A and B. For TiO_2_ samples, a peak at 690 cm ⁻ ¹ was observed, corresponding to the Ti-O stretching band, a characteristic feature of TiO_2_ [42]. For ZnO samples, the absorption band around 490 cm ⁻ ¹ was associated with the stretching mode of the Zn-O bond [43]. The immobilization of HRP on TiO_2_ led to the appearance of a peak around 1200 cm ⁻ ¹, which corresponds to the C-N vibration band, typically observed when proteins/enzymes are immobilized on TiO_2_. The identical C-N peak also appeared for the ZnO-HRP sample [42]. Additionally, the C = O peak (Amide I) from the HRP enzyme was observed around 1650 cm ⁻ ¹, along with the N-H stretch peak around 3400 cm ⁻ ¹ [44,45], thus verifying the immobilization of HRP onto TiO_2_ and ZnO.

### Impact of pH and temperature on the stability of both free and immobilized HRP

**Fig 6:**
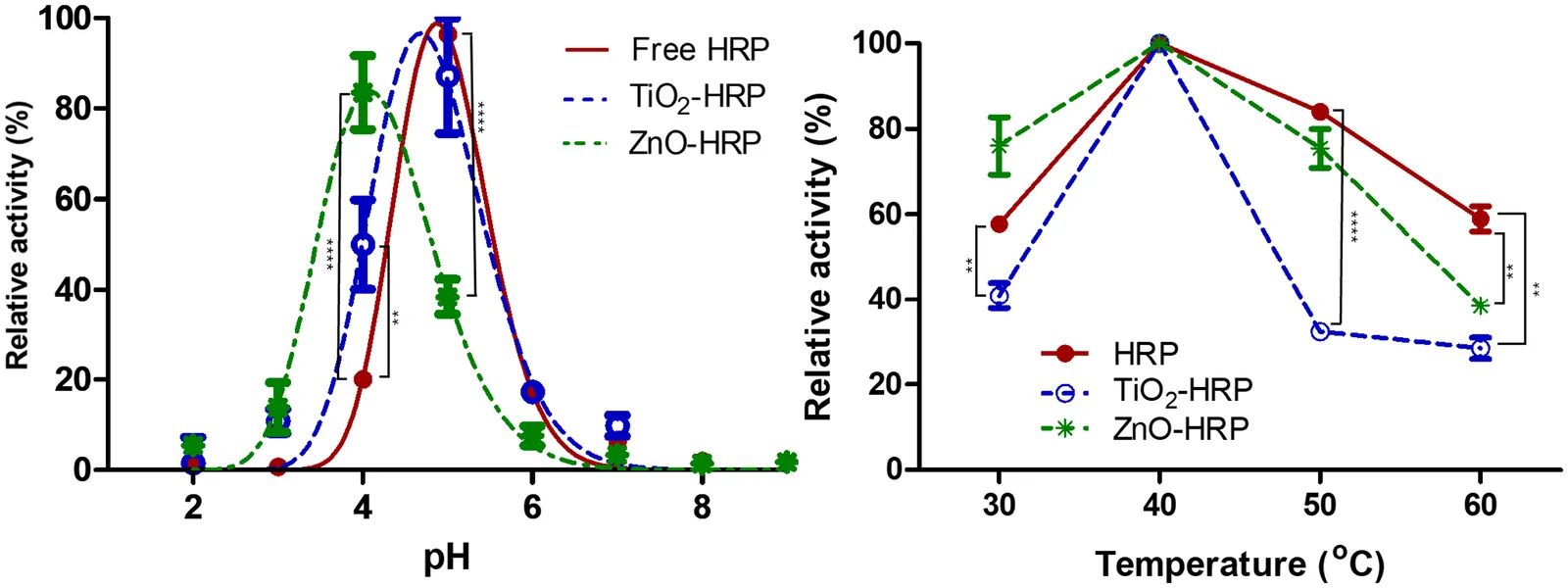
Comparative pH and temperature profiles of free and immobilized HRP. A. pH profile of free and immobilized HRP, B. Temperature profile of free and immobilized HRP. Each experiment was done in triplicates and the error bars represent ± one standard deviation (SD). The asterisk (*) shows the significant difference, where p < 0.05 (*), p < 0.01 (**), p < 0.001 (***), and p < 0.0001 (****).

Enzyme-based reactions are strongly pH-dependent. To establish the optimal pH for HRP enzymes immobilized on photocatalysts TiO_2_ and ZnO, we conducted experiments across a pH range of 2–9 for both free and immobilized HRP enzymes while keeping all other parameters constant (see Fig 6A). The results indicated that the optimal pH for both free and TiO_2_-immobilized HRP was 5. This observation is consistent with previous reports indicating that peroxidase-type catalytic activity is strongly pH-dependent and generally favored under mildly acidic conditions [46]. Although the operable pH range remained unchanged for the free enzyme and the immobilized HRP, there was a slight improvement at pH 4 for the immobilized HRP, with activity increasing from 20% in the free enzyme to 50% in the immobilized version. Interestingly, the immobilization of HRP on ZnO caused a minor shift in its optimal pH to 4, a phenomenon commonly observed when enzymes are immobilized on solid supports [47].

Since temperature also significantly affects enzyme activity, we evaluated whether immobilizing HRP on TiO_2_ and ZnO could extend the operable temperature range compared to free HRP. Temperature experiments were performed from 30°C to 60°C (Fig 6B). The results showed that immobilized HRP exhibited similar thermostability to free HRP, with peak activity at 40°C. However, a marked decline in activity was observed at higher temperatures. TiO_2_-HRP displayed a sharp drop in activity after 40°C, demonstrating poor thermal stability with immobilization. In contrast, ZnO-HRP maintained higher activity at higher temperatures, indicating improved enzyme stability. These findings agree with earlier studies showing that HRP exhibits high catalytic activity within the 30–40 °C range before gradually decreasing at higher temperatures [48].

### Degradation of 21 EPs using hybrid biocatalysts (TiO_2_-HRP and ZnO-HRP)

The degradation of the 21 EPs was also examined using immobilized HRP on TiO_2_ and ZnO, with and without the addition of HOBT as a redox mediator. Table 3 provides a summary of the results obtained from the TiO_2_ -HRP treatment of the 21 EPs. The results showed that the TiO_2_-HRP hybrid biocatalyst alone effectively degraded 81.7% of caffeic acid and 60.1% of cimetidine. Moderate degradation rates were achieved for roxithromycin, caffeine, and MBT, with degradation percentages of 46.2%, 43.8%, and 24.1%, respectively. Four EPs showed degradation rates ranging from 16% to 10%, including furosemide, meloxicam, prometryn, and SMX. The remaining 12 EPs were poorly degraded by the TiO_2_ -HRP hybrid biocatalyst, with degradation percentages of less than 10%. These EPs included lincomycin-HCl, phenytoin, DEET, norfloxacin, venlafaxine-HCl, trimethoprim, MCPA, atenolol, fluometuron, ibuprofen, and hydrochlorothiazide. The addition of HOBT improved the degradation efficiency of the TiO_2_ -HRP biocatalyst for some pollutants. For example, the degradation of cimetidine increased from 60.1% to 66.7% with HOBT. An enhanced degradation of MBT was observed, with the degradation percentage rising from 46.2% to 55.1%. Additionally, the degradation of MCPA, atenolol, and ibuprofen increased from 3.6% to 16%, 1.6% to 14.6%, and 0% to 16.7%, respectively. However, the addition of HOBT did not significantly affect the degradation of many pollutants, such as furosemide (from 16.2% to 16.8%) and phenytoin (from 9% to 9.8%). On the other hand, the addition of HOBT inhibited the degradation of several pollutants. For instance, the degradation of caffeic acid was fully inhibited by HOBT. Moreover, HOBT strongly inhibited meloxicam degradation and reduced the degradation of roxithromycin, caffeine, prometryn, and lincomycin-HCl. The degradation of the same set of EPs was also tested using the ZnO-HRP hybrid biocatalyst, with and without HOBT. Table 4 reports the results. The ZnO-HRP hybrid biocatalyst was able to effectively degrade MBT alone, with a degradation percentage of 86%. Additionally, it degraded 58.2% of roxithromycin, 32.7% of caffeine, and 30.2% of caffeic acid. The remaining EPs were poorly degraded under these conditions, generally showing low or negligible degradation. The addition of HOBT significantly improved enzyme-mediated degradation, leading to total degradation of caffeic acid. It also boosted the degradation of MBT from 86% to 90%. Furthermore, the use of the redox mediator enabled the degradation of 21.4% of hydrochlorothiazide, which was resistant to degradation by the hybrid biocatalyst alone. In contrast, the addition of HOBT caused a slight decrease in the degradation of roxithromycin and caffeine.

**Table 3:** Degradation of 21 EPs treated with TiO_2_-HRP and TiO_2_-HRP + HOBT.

Emerging Pollutants	TiO_2_-HRP %Degradation	TiO_2_-HRP + HOBT %Degradation
Caffeic acid	81.7 ± 1.4	0.0 ± 0.1
Cimetidine	60.1 ± 3.8	66.7 ± 3.4
MBT	46.2 ± 0.9	55.1 ± 6.7
Roxithromycin	43.8 ± 9.9	4.6 ± 3.8
Caffeine	24.1 ± 7.2	8.5 ± 9.7
Furosemide	16.2 ± 3.1	16.8 ± 3.9
Meloxicam	15.1 ± 9.8	0.0 ± 0.1
Prometryn	14.7 ± 5.4	9.2 ± 0.2
SMX	10.4 ± 4.3	12.2 ± 1.2
Lincomycin-HCl	9.5 ± 3.5	4.4 ± 4.7
Phenytoin	9.0 ± 4.4	9.8 ± 6.8
DEET	8.8 ± 2.7	17.8 ± 1.8
Norfloxacin	7.7 ± 5.7	11.7 ± 5.9
Venlafaxine-HCl	6.5 ± 2.3	12.7 ± 5.2
Trimethoprim	5.2 ± 3.4	8.7 ± 2.2
Thiabendazole	4.4 ± 3.2	8.1 ± 2.5
MCPA	3.6 ± 4.7	15.9 ± 3.9
Atenolol	1.6 ± 8.7	14.6 ± 9.6
Fluometuron	0.0 ± 0.1	1.9 ± 5.0
Ibuprofen	0.0 ± 0.1	16.7 ± 4.9
Hydrochlorothiazide	0.0 ± 0.1	8.7 ± 4.4

**Table 4:** Degradation of 21 EPs treated with ZnO-HRP and ZnO-HRP + HOBT.

Emerging Pollutants	ZnO-HRP %Degradation	ZnO-HRP + HOBT %Degradation
MBT	86.0 ± 2.5	90.0 ± 3.3
Roxithromycin	58.2 ± 7.8	55.4 ± 4.4
Caffeine	32.7 ± 6.2	30.6 ± 5.9
Caffeic acid	30.2 ± 5.5	100.0 ± 0.0
Ibuprofen	11.6 ± 8.1	18.0 ± 4.9
Norfloxacin	10.9 ± 5.4	13.3 ± 5.1
Prometryn	9.8 ± 2.2	11.7 ± 3.3
Furosemide	9.1 ± 1.6	8.4 ± 2.5
SMX	7.7 ± 1.0	8.8 ± 7.9
Atenolol	6.1 ± 5.2	7.2 ± 3.3
Lincomycin-HCl	5.8 ± 7.4	9.0 ± 6.6
Meloxicam	5.6 ± 7.7	13.7 ± 2.5
Cimetidine	5.3 ± 4.2	10.3 ± 0.6
Thiabendazole	4.3 ± 4.4	3.9 ± 2.7
Venlafaxine-HCl	3.8 ± 5.5	5.2 ± 2.2
DEET	3.6 ± 0.9	14.1 ± 1.7
Trimethoprim	3.3 ± 2.4	3.2 ± 4.4
Fluometuron	2.6 ± 8.1	10.7 ± 7.1
Hydrochlorothiazide	2.2 ± 3.0	21.4 ± 7.1
Phenytoin	0.0 ± 4.3	6.0 ± 3.4
MCPA	0.0 ± 0.0	2.8 ± 5.3

A comparison with our previous work, where SBP was immobilized on TiO_2_ and ZnO, reveals significant differences in the catalytic performance of both enzymes [49]. HRP demonstrates a higher degradation efficiency for certain pollutants, while SBP is more effective for others. For instance, TiO_2_-HRP degraded approximately 43.8% of roxithromycin, whereas TiO_2_-SBP achieved only 24.4%. Similarly, ZnO-HRP exhibited superior degradation of caffeine (32.7%), while ZnO-SBP showed almost no activity (0.2%). However, SBP outperformed HRP in the degradation of norfloxacin, with TiO_2_-SBP achieving 45.1% degradation compared to just 7.7% for TiO_2_-HRP. These findings emphasize the distinct catalytic performance of each enzyme, thereby demonstrating the limitations of a one-size-fits-all approach in biocatalysis. Selecting the appropriate enzyme-support combination is significant for optimizing pollutant degradation, underscoring the need for a more tailored strategy in environmental and water remediation.

### Comparison between free and immobilized HRP enzyme

The capability of the hybrid biocatalyst was evaluated against the free HRP enzyme concerning the degradation of 21 EPs. Tables 5, 6, 7 and 8 summarize all the data collected from treating the 21 EPs with free HRP and immobilized HRP on TiO_2_ and ZnO, both in the presence and absence of the redox mediator, HOBT. Several pollutants, including Roxithromycin, Cimetidine, and Caffeine, showed higher degradation rates when treated with the immobilized enzyme systems (TiO_2_-HRP or ZnO-HRP) compared to the free HRP enzyme. Notably, some pollutants, such as Roxithromycin, were poorly degraded by free HRP but exhibited significant degradation when treated with the immobilized HRP systems. Specifically, ZnO-HRP increased degradation from 0.5% to 58.2%. For cimetidine, degradation increased from 0.3% with free HRP to 60.1% with TiO_2_-HRP in the absence of HOBT, and from 9.0% with HRP + HOBT to 66.7% with TiO_2_-HRP + HOBT. Similarly, Caffeine, a relatively recalcitrant pollutant for free HRP (14.9%), showed notable improvement when treated with TiO_2_-HRP (24.1%) and ZnO-HRP (32.7%). The enhanced degradation of several EPs by the immobilized HRP could be attributed to the synergistic effect of the radicals produced by both the HRP enzyme and the photocatalysts, as reported previously [50,51]. Interestingly, some EPs, such as Meloxicam, SMX, and Furosemide, were better degraded by free HRP than by the immobilized enzyme systems. Free HRP achieved nearly complete degradation for all three EPs, while TiO₂-HRP and ZnO-HRP systems resulted in less than 20% degradation. This may be explained by the fact that when glutaraldehyde is used for enzyme immobilization, it can partially denature or conformationally constrain the enzyme, leading to a loss of enzyme activity [52]. Some pollutants, such as MBT and Caffeic acid, exhibited similar degradation rates when using either free or immobilized HRP, with degradation rates exceeding 80%. Of the 21 EPs tested, eight pollutants—specifically Prometryn, Ibuprofen, Norfloxacin, Fluometuron, Atenolol, DEET, Phenytoin, and Thiabendazole—were resistant to degradation by both free and immobilized HRP, showing degradation rates of less than 20%.

**Table 5:** Statistical comparison (t-test) between HRP and TiO_2_-HRP treatments showing mean ± SD and significance for pollutant degradation efficiency. The asterisk (*) shows the significant difference, where p < 0.05 (*), p < 0.01 (**), p < 0.001 (***), and p < 0.0001 (****). “N.S.” denotes not significant.

Emerging Pollutants	HRP % Degradation	TiO_2_-HRP % Degradation	Significance (*)
MBT	100 ± 0.0	46.2 ± 0.9	****
Meloxicam	99.5 ± 0.1	15.1 ± 9.8	**
Caffeic acid	98.6 ± 1.4	81.7 ± 1.4	***
Furosemide	45.8 ± 3.0	16.2 ± 3.1	***
SMX	38.9 ± 2.0	10.4 ± 4.3	**
MCPA	20 ± 8.1	3.6 ± 4.7	N.S.
Prometryn	19.2 ± 5.3	14.7 ± 5.4	N.S.
Ibuprofen	13.2 ± 7.9	0.0 ± 0.1	N.S.
Hydrochlorothiazide	12.2 ± 2.5	0.0 ± 0.1	*
Thiabendazole	3.8 ± 3.7	4.4 ± 3.2	N.S.
Venlafaxine-HCl	3.3 ± 7.2	6.5 ± 2.3	N.S.
Phenytoin	3.1 ± 1.5	9.0 ± 4.4	N.S.
Roxithromycin	0.5 ± 7.2	43.8 ± 9.9	**
Cimetidine	0.3 ± 4.8	60.1 ± 3.8	***
Lincomycin-HCl	0.0 ± 0.0	9.5 ± 3.5	*
Norfloxacin	0.0 ± 0.0	7.7 ± 5.7	N.S.
Trimethoprim	0.0 ± 0.0	5.2 ± 3.4	N.S.
Atenolol	0.0 ± 0.0	1.6 ± 8.7	N.S.
Fluometuron	0.0 ± 0.0	0.0 ± 0.1	N.S.
Caffeine	0.0 ± 0.0	24.1 ± 7.2	*
DEET	0.0 ± 0.0	8.8 ± 2.7	*

**Table 6:** Statistical comparison (t-test) between HRP + HOBT and TiO_2_-HRP + HOBT treatments showing mean ± SD and significance for pollutant degradation efficiency. The asterisk (*) shows the significant difference, where p < 0.05 (*), p < 0.01 (**), p < 0.001 (***), and p < 0.0001 (****).“N.S.” denotes not significant.

Emerging Pollutants	HRP + HOBT % Degradation	TiO₂-HRP + HOBT % Degradation	Significance
MBT	100 ± 0.0	55.1 ± 6.7	**
Meloxicam	99.6 ± 0.1	0.0 ± 0.1	****
Caffeic acid	100 ± 0.0	0.0 ± 0.1	****
Furosemide	100 ± 0.0	16.8 ± 3.9	***
SMX	99 ± 0.1	12.2 ± 1.2	****
MCPA	17.4 ± 8.7	15.9 ± 3.9	N.S.
Prometryn	13.4 ± 6.2	9.2 ± 0.2	N.S.
Ibuprofen	12.1 ± 4.0	16.7 ± 4.9	N.S.
Hydrochlorothiazide	24.8 ± 4.2	8.7 ± 4.4	*
Thiabendazole	13.2 ± 2.3	8.1 ± 2.5	N.S.
Venlafaxine-HCl	20.1 ± 8.8	12.7 ± 5.2	N.S.
Phenytoin	2.6 ± 3.7	9.8 ± 6.8	N.S.
Roxithromycin	0.0 ± 0.0	4.6 ± 3.8	N.S.
Cimetidine	9.0 ± 3.3	66.7 ± 3.4	****
Lincomycin-HCl	36.9 ± 8.1	4.4 ± 4.7	**
Norfloxacin	12.7 ± 4.1	11.7 ± 5.9	N.S.
Trimethoprim	21.5 ± 4.7	8.7 ± 2.2	*
Atenolol	3.1 ± 3.7	14.6 ± 9.6	N.S.
Fluometuron	17.1 ± 4.0	1.9 ± 5.0	*
Caffeine	14.9 ± 3.6	8.5 ± 9.7	N.S.
DEET	6.0 ± 2.5	17.8 ± 1.8	**

**Table 7:** Statistical comparison (t-test) between HRP and ZnO-HRP treatments showing mean ± SD and significance for pollutant degradation efficiency. The asterisk (*) shows the significant difference, where p < 0.05 (*), p < 0.01 (**), p < 0.001 (***), and p < 0.0001 (****).“N.S.” denotes not significant.

Emerging Pollutant	HRP % Degradation	ZnO-HRP % Degradation	Significance (*)
MBT	100 ± 0.0	86.0 ± 2.5	***
Meloxicam	99.5 ± 0.1	5.6 ± 7.7	***
Caffeic acid	98.6 ± 1.4	30.2 ± 5.5	***
Furosemide	45.8 ± 3.0	9.1 ± 1.6	***
SMX	38.9 ± 2.0	7.7 ± 1.0	***
MCPA	20.0 ± 8.1	0.0 ± 0.0	N.S.
Prometryn	19.2 ± 5.3	9.8 ± 2.2	*
Ibuprofen	13.2 ± 7.9	11.6 ± 8.1	N.S.
Hydrochlorothiazide	12.2 ± 2.5	2.2 ± 3.0	N.S.
Thiabendazole	3.8 ± 3.7	4.3 ± 4.4	N.S.
Venlafaxine-HCl	3.3 ± 7.2	3.8 ± 5.5	N.S.
Phenytoin	3.1 ± 1.5	0.0 ± 4.3	N.S.
Roxithromycin	0.5 ± 7.2	58.2 ± 7.8	***
Cimetidine	0.3 ± 4.8	5.3 ± 4.2	N.S.
Lincomycin-HCl	0.0 ± 0.0	5.8 ± 7.4	N.S.
Norfloxacin	0.0 ± 0.0	10.9 ± 5.4	N.S.
Trimethoprim	0.0 ± 0.0	3.3 ± 2.4	N.S.
Atenolol	0.0 ± 0.0	6.1 ± 5.2	N.S.
Fluometuron	0.0 ± 0.0	2.6 ± 8.1	N.S.
Caffeine	0.0 ± 0.0	32.7 ± 6.2	***
DEET	0.0 ± 0.0	3.6 ± 0.9	***

Recent studies have demonstrated the strong potential of immobilized enzymes for pollutant degradation. For instance, laccase immobilized on amino-functionalized PMMA-reinforced graphene nanomaterial achieved 84.5% degradation of bisphenol A after five cycles, highlighting its effectiveness toward removing phenolic contaminants [53]. Similarly, laccase immobilized on chitosan– alginate–Fe_3_O_4_ magnetic composites exhibited high immobilization efficiency (91%) and enhanced catalytic performance [54], while β-glucosidase immobilized on amidoximated acrylic fabric integrated with Fe_3_O_4_ nanoparticles achieved an 89% yield [55]. These findings support the growing evidence that enzyme immobilization enhances catalytic efficiency for pollutant removal. The comparable degradation efficiencies observed for TiO_2_–HRP and ZnO–HRP hybrid biocatalysts in this work further confirm the robustness of immobilized oxidoreductases for environmental remediation applications.

**Table 8:** Statistical comparison (t-test) between HRP + HOBT and ZnO-HRP + HOBT treatments showing mean ± SD and significance for pollutant degradation efficiency. The asterisk (*) shows the significant difference, where p < 0.05 (*), p < 0.01 (**), p < 0.001 (***), and p < 0.0001 (****).“N.S.” denotes not significant.

Emerging Pollutant	HRP + HOBT % Degradation	ZnO-HRP + HOBT % Degradation	Significance (*)
MBT	100 ± 0.0	90.0 ± 3.3	**
Meloxicam	99.6 ± 0.1	13.7 ± 2.5	****
Caffeic acid	100 ± 0.0	100 ± 0.0	N.S.
Furosemide	100 ± 0.0	8.4 ± 2.5	****
SMX	99.0 ± 0.1	8.8 ± 7.9	***
MCPA	17.4 ± 8.7	2.8 ± 5.3	N.S.
Prometryn	13.4 ± 6.2	11.7 ± 3.3	N.S.
Ibuprofen	12.1 ± 4.0	18.0 ± 4.9	N.S.
Hydrochlorothiazide	24.8 ± 4.2	21.4 ± 7.1	N.S.
Thiabendazole	13.2 ± 2.3	3.9 ± 2.7	*
Venlafaxine-HCl	20.1 ± 8.8	5.2 ± 2.2	N.S.
Phenytoin	2.6 ± 3.7	6.0 ± 3.4	N.S.
Roxithromycin	0.0 ± 0.0	55.4 ± 4.4	***
Cimetidine	9.0 ± 3.3	10.3 ± 0.6	N.S.
Lincomycin-HCl	36.9 ± 8.1	9.0 ± 6.6	**
Norfloxacin	12.7 ± 4.1	13.3 ± 5.1	N.S.
Trimethoprim	21.5 ± 4.7	3.2 ± 4.4	**
Atenolol	3.1 ± 3.7	7.2 ± 3.3	N.S.
Fluometuron	17.1 ± 4.0	10.7 ± 7.1	N.S.
Caffeine	14.9 ± 3.6	30.6 ± 5.9	*
DEET	6.0 ± 2.5	14.1 ± 1.7	**

### Computational and docking studies: Binding to emerging pollutants and correlation with their degradation

**Fig 7:**
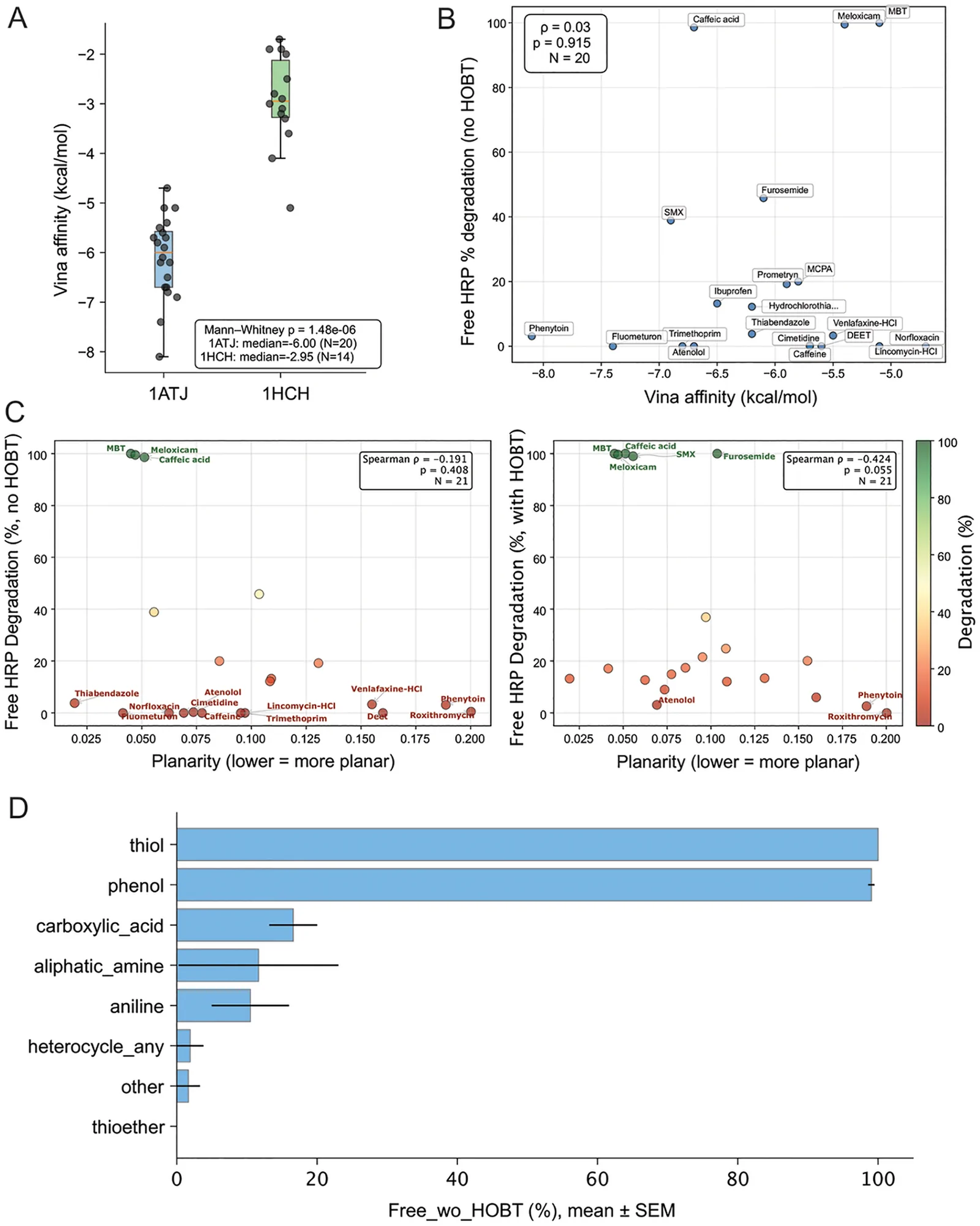
Docking, molecular planarity, and functional-group analyses reveal structural factors influencing pollutant degradation by free HRP. A) Distribution of Vina scores by receptor (free HRP models). Boxplots comparing AutoDock Vina scores for the resting ferric (1ATJ) and reactive Compound I (1HCH) forms of HRP. The 1ATJ model exhibits more favorable Vina scores (Mann–Whitney p < 1 × 10^−5^). B) Free HRP degradation versus docking score (1ATJ receptor). Scatterplot showing experimental degradation percentages plotted against calculated Vina scores. No significant correlation (ρ = 0.03, p = 0.92) was observed. C) Planarity versus degradation efficiency for free HRP. Left: free HRP without HOBT (ρ = –0.19, p = 0.41); right: free HRP with HOBT (ρ = –0.42, p = 0.055). More planar ligands such as MBT, caffeic acid, and meloxicam were associated with higher degradation levels. D. Functional group contribution to pollutant degradation. Bar chart summarizing mean degradation percentages grouped by dominant functional class under free HRP (no HOBT) conditions.

In order to explore potential correlation between HRP and EP binding and their degradation, docking simulations were performed using two HRP crystal structures: the resting ferric form (1ATJ) and the reactive Compound I state (1HCH). The two HRP structures displayed markedly different docking score distributions. The resting-state 1ATJ model produced more favorable Vina scores (median = –5.9 kcal mol^-1^) than the oxidized 1HCH form (median = –3.0 kcal mol^-1^, Mann–Whitney p < 1 × 10^−5^), consistent with differences in pocket geometry between the structures (Fig 7A). The distribution of Vina scores and their relationship with experimental degradation are shown in Fig 7B and S3 Fig. When the predicted Vina scores were compared with experimental degradation by free HRP, no statistically significant correlation was observed for either receptor. For 1ATJ, the correlation between docking score and degradation was weak (ρ = 0.03, p = 0.92), and for 1HCH it remained modest (ρ = –0.48, p = 0.085). These findings support that static docking scores alone cannot fully explain the observed degradation efficiencies.

To simulate the restricted substrate access caused by immobilization, docking was repeated using the occluded grid centered on the heme Fe atom. This configuration produced higher (less favorable) Vina scores overall, consistent with limited pocket accessibility. In the occluded state, the TiO_2_-HRP model exhibited a moderate inverse relationship between predicted Vina score and experimental degradation (ρ = –0.41, p = 0.18, N = 12) (S4 Fig). Compounds able to retain valid poses under these constraints, such as caffeic acid, MBT, and cimetidine, also showed higher degradation in experiments, consistent with accessibility contributing to activity under the tested conditions.

Planarity, quantified as the ratio of the smallest to the largest eigenvalue of the atomic coordinate covariance matrix, displayed a weak inverse relationship with degradation in free HRP without mediator (ρ = –0.19, p = 0.41) but a stronger correlation when HOBT was present (ρ = –0.42, p = 0.055, N = 21) (Fig 7C). The most planar compounds, MBT, caffeic acid, and meloxicam, were also among the most easily degraded. A more direct statistical comparison between highly degradable (> 90%) and poorly degradable (≤ 20%) compounds revealed a significant difference in mean planarity (0.0478 vs 0.1046; Mann–Whitney p = 0.0475) (S5 Fig), corresponding to a 2.2-fold difference in molecular flatness. This supports an association between molecular planarity and favorable orientation for electron transfer at the heme site.

Grouping the pollutants by major functional class revealed chemical trends (Fig 7D). Thiol- and phenol-containing compounds showed the highest mean degradation under free HRP conditions without HOBT, reflecting the high redox susceptibility of both sulfur- and hydroxyl-bearing substrates. Carboxylic acids and heterocycles were generally less reactive, while aliphatic amines exhibited only moderate degradation. To test whether degradation efficiency correlates with substrate approach to the catalytic center, minimum Fe–ligand distances were extracted from docked poses. Across the compound panel, distances averaged 6.3 ± 0.6 Å, showing no clear dependence on degradation category. The correlation between degradation and Fe distance was weak (ρ = –0.08, p = 0.74) (S6 Fig), suggesting that catalytic reactivity depends more on orientation and molecular geometry than on absolute distance to the heme iron. Correlation analysis of RDKit descriptors indicated that small, moderately hydrophobic molecules with accessible polar groups tended to be more degradable, though no single descriptor governed activity (S7 Fig).

Overall, the docking analyses revealed that while HRP can bind a wide range of emerging pollutants, the predicted static docking scores alone do not reliably explain their experimental degradation trends. Instead, the results highlight the importance of enzyme accessibility, substrate geometry, and electronic structure in determining degradation outcomes. The reduced binding in the occluded model is consistent with steric hindrance limiting substrate access to the heme site, while the observed association with molecular planarity points to the role of favorable orientation for electron transfer. Functional group analysis further emphasizes that redox-active moieties such as thiols and phenols enhance reactivity, whereas bulky or less planar scaffolds hinder degradation. Collectively, these findings suggest that pollutant degradation by HRP is governed by an interplay of structural accessibility, molecular planarity, and redox potential, rather than by docking score alone. These insights can inform the rational design of improved immobilization matrices and HRP-based catalytic systems for pollutant removal.

To provide mechanistic context, docking results were interpreted relative to the HRP catalytic architecture rather than as docking score rankings alone. In HRP, substrate oxidation occurs at the heme center and is strongly influenced by substrate approach geometry and orientation within the distal pocket (including residues such as Arg38, Phe41, and His42), which can modulate electron transfer feasibility. Therefore, this docking analysis was designed to quantify access to the catalytic environment (Fe proximity), residue-level interaction patterns and active-site contact topology, and then compare these structural descriptors with experimental degradation behavior.

To support this analysis, S6 Table summarizes per-ligand secondary-structure composition of contact residues across free and occluded HRP docking conditions, including residue counts and helix, beta, turn, and coil percentages.

**Fig 8:**
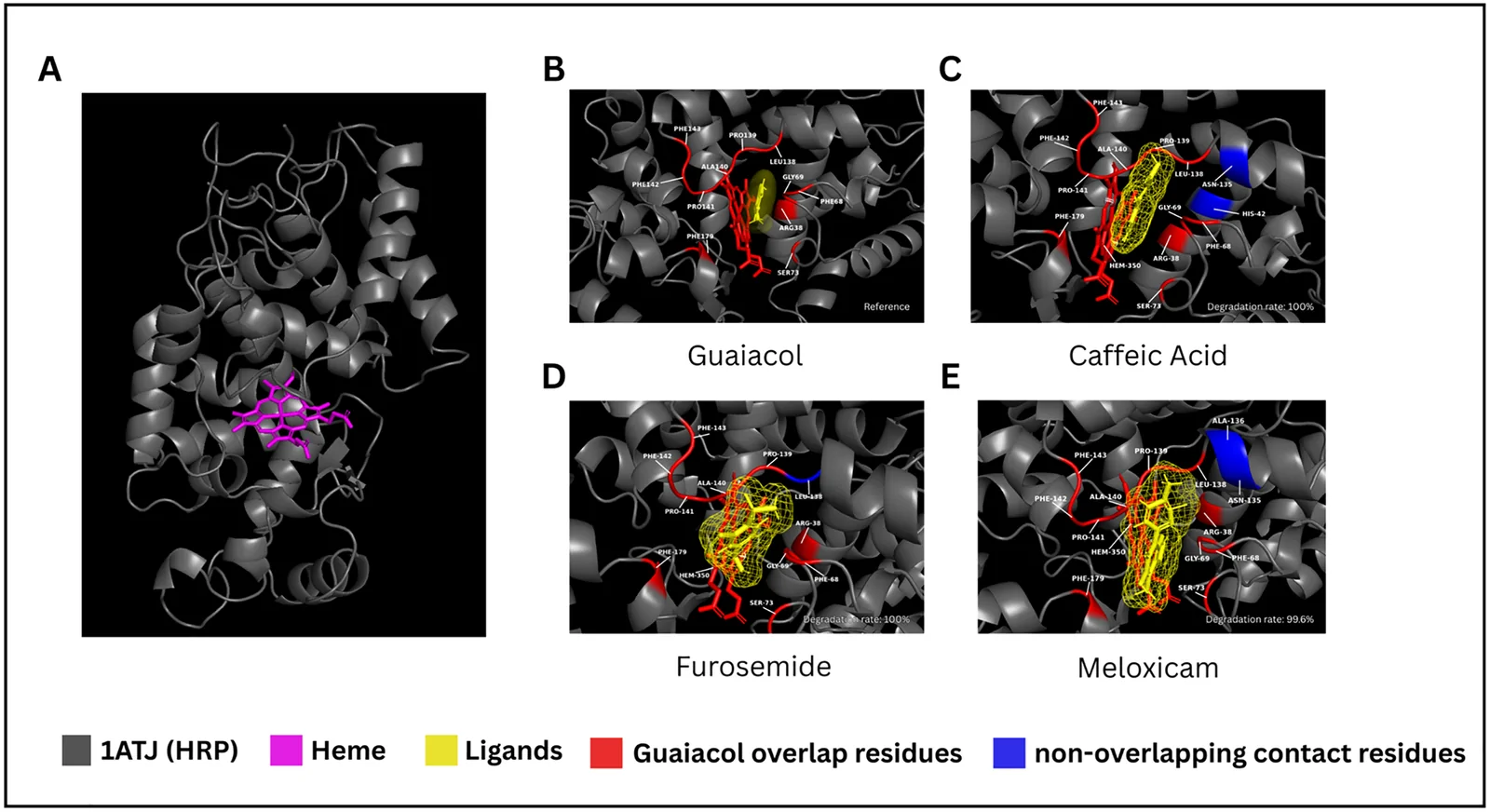
Representative structural overlays in 1ATJ showing guaiacol overlap residues and non-overlapping contact residues for selected degradable ligands. A. 3D structure of HRP shown in gray cartoon representation, highlighting the heme prosthetic group in magenta located within the catalytic pocket. B. Binding pose of the reference substrate guaiacol (yellow) within the HRP active site. Residues interacting with guaiacol are depicted in red. C-E. Docking conformations of selected ligands: caffeic acid (C), furosemide (D), and meloxicam (E), shown in yellow. Amino acid residues overlapping with guaiacol-binding interactions are highlighted in red, while additional non-overlapping contact residues are shown in blue. The heme group is represented as HEM-350. Experimental degradation percentages are indicated in each panel.

For structural context and visual validation, representative 3D structural models have been generated (Fig 8). Substantial overlap was observed between guaiacol and selected degradable compounds (caffeic acid, furosemide, and meloxicam) within the distal heme pocket. Shared residues included Arg38, Phe68, Gly69, Ser73, Leu138, Pro139, Ala140, Pro141, Phe142, Phe143, and Phe179. These conserved interactions indicate that responsive substrates can occupy a similar catalytic environment near the heme center, supporting their compatibility with HRP-mediated oxidation.

Further secondary structural analyses under all evaluated scenarios (free vs. occluded configurations, with and without HOBT) as well as comparisons between low degradation and high degradation groups have been performed yielding no statistically significant trends.

### Evaluating the photocatalytic performance of hybrid biocatalysts

**Fig 9:**
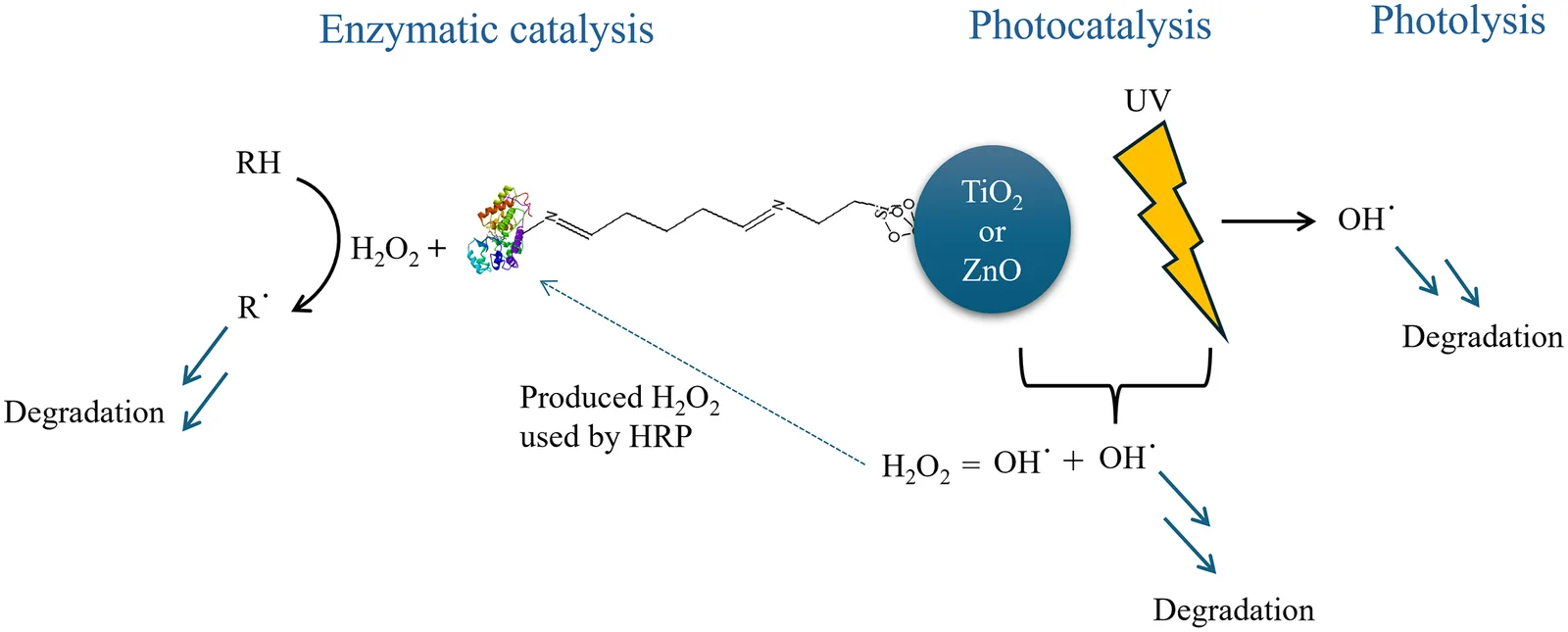
Hybrid peroxidase-photocatalyst system.

**Fig 10:**
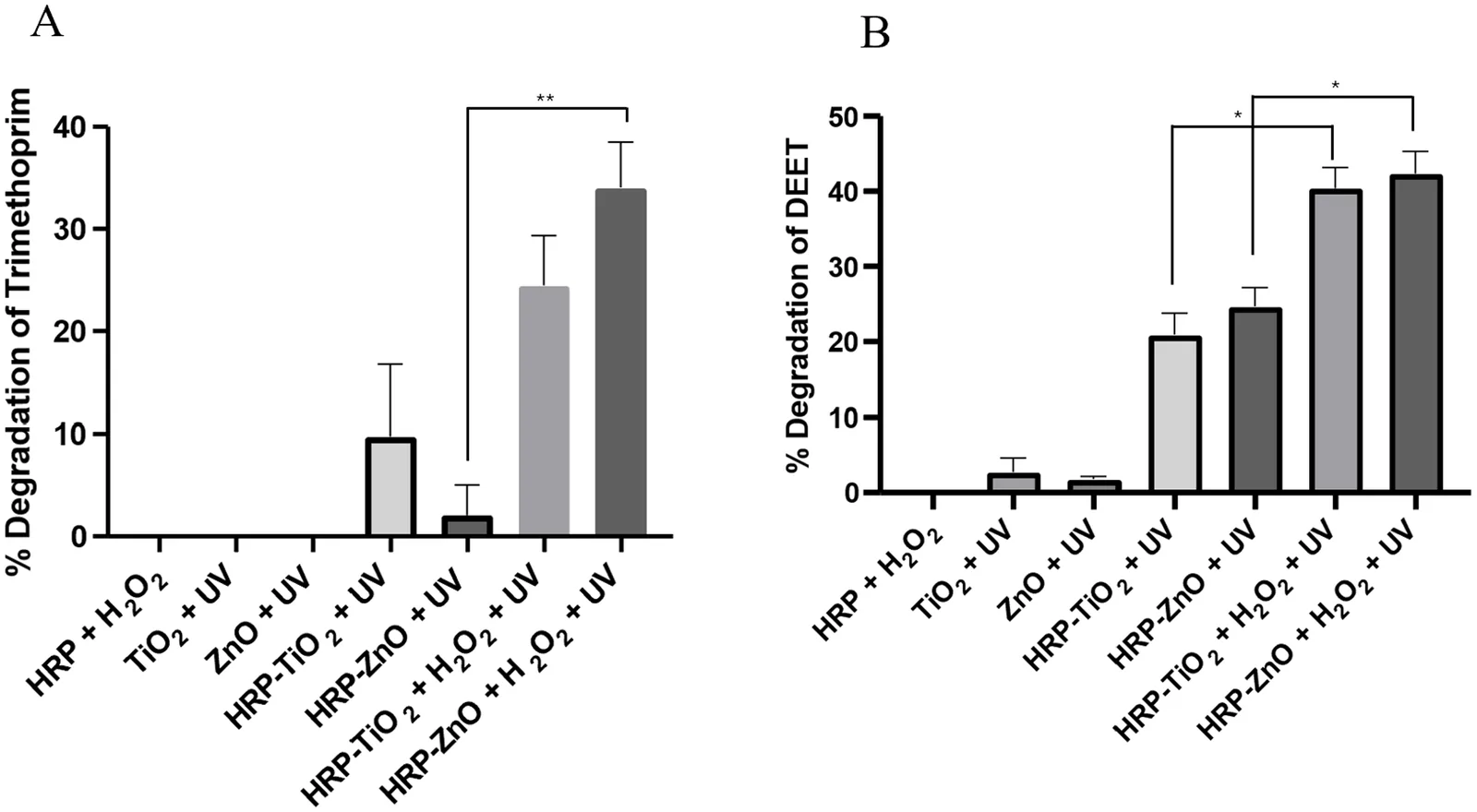
Percentage degradation of A. Trimethoprim and B. DEET. Each experiment was done in duplicate and the error bars represent ± one standard deviation (SD). The asterisk (*) shows the significant difference, where p < 0.05 (*), p < 0.01 (**), p < 0.001 (***), and p < 0.0001 (****).

Both solid supports used for HRP immobilization in this work (TiO_2_ and ZnO) are photocatalytic. Therefore, combining the remediation capabilities of the TiO_2_/ZnO photocatalysts with the HRP enzyme could potentially result in a powerful hybrid peroxidase-photocatalyst system (Fig 9). As illustrated in Fig 9 such as recyclability, UV radiation exposure induces the generation of reactive OH radicals by TiO_2_ or ZnO photocatalysts, which can degrade EPs. Additionally, this process generates H_2_O_2_, which can be utilized by HRP to further degrade EPs or be broken down by UV light to create more reactive radicals. Consequently, the combined action of enzymatic catalysis, photocatalysis, and photolysis could, in theory, facilitate a swifter and more efficient degradation of EPs. To test this hypothesis, two EPs (trimethoprim and DEET) were selected as model pollutants (Fig 10A and 10B). When either HRP alone or the neat photocatalysts (TiO_2_ or ZnO) were used, no degradation of the pollutants was observed. However, exposure of the hybrid biocatalysts (TiO_2_-HRP and ZnO-HRP) to UV light resulted in slight degradation. Specifically, TiO_2_-HRP + UV achieved approximately 10% degradation of trimethoprim, while both TiO_2_-HRP + UV and ZnO-HRP + UV achieved around 20% degradation of DEET. Interestingly, the addition of H_2_O_2_ significantly enhanced the degradation rates. For trimethoprim, TiO_2_-HRP + H₂O₂ + UV achieved around 30% degradation, while for DEET, both TiO_2_-HRP + H_2_O_2_ + UV and ZnO-HRP + H_2_O_2_ + UV achieved approximately 40% degradation. These findings confirm that immobilizing enzymes onto photocatalytic supports enhances the degradation of EPs compared to using the enzyme alone or neat photocatalysts. This improvement is attributed to the synergistic effects of enzymatic catalysis, photocatalysis, and photolysis occurring simultaneously in the reaction system. These results are consistent with previous studies. For example, a TiO_2_-HRP hybrid catalyst system degraded over 90% of 2,4-dichlorophenol within 3 hours [50]. Similarly, immobilization of HRP on ZnO nanowires/macroporous silicon dioxide composites successfully degraded 95.9% of Reactive Blue 19 and 94.3% of Acid Violet 109 dyes [56]. The current study further validates the application of hybrid peroxidase-photocatalyst systems and expands their potential to a broader panel of 21 different EPs.

## Conclusion

In this study, we successfully developed and characterized two novel hybrid biocatalysts, TiO_2_-HRP and ZnO-HRP, by immobilizing the HRP enzyme via covalent attachment onto functionalized photocatalytic supports (TiO_2_ and ZnO). The characterization using SEM, XRD, and IR confirmed the successful immobilization of HRP onto the supports, ensuring the structural and functional integrity of the hybrid systems. Our findings demonstrate that while HRP alone can degrade certain EPs, the integration of photocatalytic supports significantly enhances its catalytic performance. Among the 21 tested EPs, HRP-based treatment showed compound-dependent degradation, with complete or near-complete degradation of MBT (100%), meloxicam (99.5%), and caffeic acid (98.6%), partial degradation of prometryn (19.2%) and MCPA (20%), and no degradation of fluometuron and atenolol. Importantly, this study demonstrates for the first time that immobilizing HRP on TiO_2_ and ZnO markedly enhances the degradation of previously recalcitrant pollutants, underscoring the novelty of the hybrid system. Notably, the addition of HOBT selectively influenced the degradation process, enhancing several pollutants while inhibiting others. For example, HOBT increased lincomycin-HCl degradation from 0% to 36.9% and trimethoprim degradation from 0% to 21.5%, whereas prometryn degradation decreased from 19.2% to 13.4% in the presence of HOBT. Pollutants including roxithromycin, cimetidine, and caffeine, displayed markedly higher degradation rates with the hybrid biocatalysts compared to the free HRP enzyme or neat photocatalysts. Specifically, roxithromycin degradation increased from 0.5% with free HRP to 58.2% with ZnO-HRP, while cimetidine degradation increased from 0.3% with free HRP to 60.1% with TiO₂-HRP in the absence of HOBT, and from 9.0% with HRP + HOBT to 66.7% with TiO₂-HRP + HOBT. This pollutant-specific enhancement highlights the synergistic interplay between enzymatic catalysis and photocatalysis, enabling effective degradation of compounds that resist both free HRP and neat photocatalysts. Together, these results confirm that integrating enzymatic and photocatalytic mechanisms can achieve more effective and versatile pollutant degradation than either process alone. Furthermore, by applying this hybrid peroxidase approach to a large panel of 21 structurally diverse pollutants, this study establishes one of the most comprehensive evaluations to date, expanding the understanding of enzyme-photocatalyst synergy across multiple compound classes. Moreover, the immobilization of HRP onto photocatalytic supports offers additional advantages, such as recyclability, and easy recovery. Finally, the integrated HRP + H_2_O_2_ + UV treatment achieved approximately 30% degradation of trimethoprim and approximately 40% degradation of DEET under the tested conditions. The findings also contribute to the growing evidence that various emerging pollutants can be degraded by free peroxidase enzymes, providing valuable insights into their broader applicability and provide a strong foundation for the development of next-generation biocatalysts. However, the degradation performance remained pollutant-specific, and the present study should be considered a controlled laboratory-scale proof of concept. Further optimization of enzyme loading, support reuse, mediator demand, real wastewater matrix effects, photoreactor design, and overall treatment cost is required before environmental-scale application can be claimed.

## Supporting information

S1 Fig The full chromatogram of 21 EPs mixture. (TIF)

S2 Fig The extracted chromatogram for 21 EPs tested. (TIF)

S3 Fig Free HRP degradation versus docking affinity (1HCH receptor). Scatterplot analogous to Figure 1B using the Compound I state. The relationship is moderately negative (ρ = –0.48, p = 0.085).
(TIF)

S4 Fig TiO₂–HRP degradation versus docking affinity (occluded model). Scatterplot correlating degradation of TiO₂-immobilized HRP with predicted binding energies under restricted pocket conditions (ρ = –0.41, p = 0.18).
(TIF)

S5 Fig Planarity comparison of good and poor degraders. Boxplot and summary statistics showing that good degraders (> 90%) are significantly more planar than poor degraders (≤ 20%) (Mann–Whitney p = 0.0475).
(TIF)

S6 Fig Fe distance versus degradation efficiency. Left: boxplot of minimum Fe–ligand distances grouped by degradation category; right: scatterplot showing no significant correlation (ρ = –0.08, p = 0.74).
(TIF)

S7 Fig Correlation heatmap between molecular descriptors and experimental degradation. Spearman correlation coefficients showing relationships among calculated physicochemical properties (molecular weight, cLogP, TPSA, HBD, HBA, aromatic rings, rotatable bonds) and degradation percentages across all HRP systems.
(TIF)

S1 Table Details of the 21 tested EPs, including supplier, catalog number, CAS number, purity, and stock Solution. (DOCX)

S2 Table Toxicity and environmental relevance of the 21 tested EPs. (DOCX)

S3 Table MRM parameters for all 21 EPs. (DOCX)

S4 Table Secondary structure correlation analysis. (DOCX)

S5 Table Secondary structure group comparison. (DOCX)

S6 Table Per-ligand secondary structure comparison in free and occluded HRP. (DOCX)
