# Supplementary material for: Immobilized horseradish peroxidase hybrid biocatalysts for degradation of pharmaceuticals and other emerging micropollutants
Source: PLoS One. 2026 Sep 2;21(9):e0355285. doi: 10.1371/journal.pone.0355285 (PMC13537563; doi:10.1371/journal.pone.0355285)
Supplement: Supplementary file 1 [file pone.0355285.s008.docx]

S1 Table: Details of the 21 tested EPs, including supplier, catalog number, CAS number, purity, and stock solution concentration.

| Emerging pollutant | Supplier | Catalog No. | CAS No. | Purity / grade | Stock-solution |
| --- | --- | --- | --- | --- | --- |
| Roxithromycin | Sigma-Aldrich | R4393 | 80214-83-1 | 95.0–102.0% / HPLC grade | 1000 ppm in methanol |
| Lincomycin-HCl | Sigma-Aldrich | 62143 | 859-18-7 | 96.0–102.0% / HPLC grade | 1000 ppm in water |
| Meloxicam | Sigma-Aldrich | PHR1799 | 71125-38-7 | Pharmaceutical secondary standard | 1000 ppm in DMSO |
| Norfloxacin | Sigma-Aldrich | N9890 | 70458-96-7 | ≥98%/ analytical standard | 1000 ppm in DMSO |
| Trimethoprim | Sigma-Aldrich | 92131 | 738-70-5 | ≥99.0% / HPLC | 1000 ppm in DMSO |
| Venlafaxine-HCl | Sigma-Aldrich | V7264 | 99300-78-4 | ≥98% / HPLC | 1000 ppm in methanol |
| Atenolol | Sigma-Aldrich | PHR1909 | 29122-68-7 | Pharmaceutical Secondary Standard | 1000 ppm in methanol |
| Sulfamethoxazole | Sigma-Aldrich | PHR1126 | 723-46-6 | Pharmaceutical Secondary Standard | 1000 ppm in methanol |
| Cimetidine | Sigma-Aldrich | PHR1075 | 51481-61-9 | Pharmaceutical secondary standard | 1000 ppm in water |
| Phenytoin | Sigma-Aldrich | PHR1139 | 57-41-0 | Pharmaceutical secondary standard | 1000 ppm in methanol |
| Prometryn | Sigma-Aldrich | 45636 | 7287-19-6 | Analytical standard | 1000 ppm in acetonitrile |
| Fluometuron | Sigma-Aldrich | 45502 | 2164-17-2 | Analytical standard | 1000 ppm in acetonitrile |
| Ibuprofen | Sigma-Aldrich | PHR1004 | 15687-27-1 | Pharmaceutical secondary standard | 1000 ppm in methanol |
| Thiabendazole | Sigma-Aldrich | 45684 | 148-79-8 | Analytical standard | 1000 ppm in methanol |
| 2-Methyl-4-chlorophenoxyacetic acid (MCPA) | Sigma-Aldrich | 45555 | 94-74-6 | Analytical standard | 1000 ppm in methanol |
| Caffeine | Sigma-Aldrich | C1778 | 58-08-2 | Sigma reference standard | 1000 ppm in water |
| DEET | Sigma-Aldrich | 36542 | 134-62-3 | Analytical standard | 1000 ppm in methanol |
| Caffeic acid | Sigma-Aldrich | C0625 | 331-39-5 | ≥98.0% / HPLC | 1000 ppm in methanol |
| Mercaptobenzothiazole | Sigma-Aldrich | CRM96051 | 149-30-4 | Certified reference material | 1000 ppm in methanol |
| Furosemide | Sigma-Aldrich | PHR1057 | 54-31-9 | Pharmaceutical secondary standard | 1000 ppm in methanol |
| Hydrochlorothiazide | Sigma-Aldrich | PHR1032 | 58-93-5 | Pharmaceutical secondary standard | 1000 ppm in methanol |
