# Supplementary material for: Immobilized horseradish peroxidase hybrid biocatalysts for degradation of pharmaceuticals and other emerging micropollutants
Source: PLoS One. 2026 Sep 2;21(9):e0355285. doi: 10.1371/journal.pone.0355285 (PMC13537563; doi:10.1371/journal.pone.0355285)
Supplement: Supplementary file 2 [file pone.0355285.s009.docx]

S2 Table: Toxicity and environmental relevance of the 21 tested EPs

| Emerging pollutant | Molecular weight (g/mol) | Compound class | Toxicity / environmental relevance | References |
| --- | --- | --- | --- | --- |
| Roxithromycin | 837.05 | Pharmaceutical | Tested on zebrafish and frog embryos; zebrafish showed malformations, heart swelling, and reduced heart rate, while frogs showed fewer malformations, mainly gut and abdominal swelling. | [1] |
| Lincomycin-HCl | 443.00 | Pharmaceutical | Caused growth inhibition in algae, with cyanobacteria being more sensitive. | [2] |
| Meloxicam | 351.40 | Pharmaceutical | Caused oxidative stress and disrupted the physiological and health status of common carp (Cyprinus carpio). | [3] |
| Norfloxacin | 319.33 | Pharmaceutical | Induced liver toxicity in common carp (Cyprinus carpio) at histological, biochemical, and transcriptional levels. | [4] |
| Trimethoprim | 290.32 | Pharmaceutical | Caused moderate toxicity in Lemna minor and slight toxicity in Aliivibrio fischeri, Escherichia coli, Daphnia magna, and Danio rerio. | [5] |
| Venlafaxine-HCl | 313.86 | Pharmaceutical | Caused neurotoxicity in zebrafish (Danio rerio), including behavioral changes, oxidative stress, and decreased AChE activity. | [6] |
| Atenolol | 266.34 | Pharmaceutical | Caused neurobehavioral toxicity in zebrafish (Danio rerio), including anxiety-like and aggressive behaviors and oxidative stress. | [7] |
| Sulfamethoxazole (SMX) | 253.28 | Pharmaceutical | Caused moderate toxicity in Lemna minor and Daphnia magna, and slight toxicity in Danio rerio. | [5] |
| Cimetidine | 252.34 | Pharmaceutical | Potential endocrine-disrupting effects may occur following long-term exposure in aquatic environments. | [8] |
| Phenytoin | 252.27 | Pharmaceutical | Triggered harmful effects in the brain of Danio rerio, resulting in anxiety-like behavior. | [9] |
| Prometryn | 241.36 | Pesticide | Caused photodamage and photosynthetic toxicity in coral symbionts, disrupting nitrate acclimation and intensifying oxidative stress in Acropora hyacinthus. | [10] |
| Fluometuron | 232.22 | Pesticide | Herbicides can pose risks to non-target organisms, including aquatic plants, fish, birds, and other wildlife. | [11] |
| Ibuprofen | 206.28 | Pharmaceutical | Caused adverse effects in aquatic organisms, including cytotoxic and genotoxic damage and oxidative stress. | [12] |
| Thiabendazole | 201.25 | Pesticide | Caused developmental toxicity in zebrafish, including morphological malformations and oxidative stress | [13] |
| 2-Methyl-4-chlorophenoxyacetic acid (MCPA) | 200.62 | Pesticide | Induced intestinal oxidative stress, inflammation, and apoptosis in loach (Misgurnus anguillicaudatus) | [14] |
| Caffeine | 194.19 | Stimulant | Caused toxic effects in Daphnia magna, including feeding inhibition, reduced growth and fecundity and altered antioxidant defenses | [15] |
| DEET | 191.27 | Personal care product | Showed possible chronic ecotoxicity concerns for non-target organisms | [16] |
| Caffeic acid | 180.16 | Phenolic compound | Inhibited soybean (Glycine max) root growth and increased root lignification. | [17] |
| Mercaptobenzothiazole (MBT) | 167.25 | Industrial chemical | Reported as an environmental concern due to its toxicity | [18] |
| Furosemide | 330.74 | Pharmaceutical | Detected in wastewater and surface water, with concentrations up to 554 ng/L | [19] |
| Hydrochlorothiazide | 297.74 | Pharmaceutical | Detected in wastewater and surface water at up to 531 ng/L | [19] |

**References**

1. Duruel HEE, Turhan DO, Ozmen M, Güngördü A. Comparative Developmental Toxicity of Roxithromycin in Danio rerio and Xenopus laevis. Journal of Applied Toxicology. 2026;46: 1963–1976. doi:10.1002/jat.70004

2. Andreozzi R, Canterino M, Giudice RL, Marotta R, Pinto G, Pollio A. Lincomycin solar photodegradation, algal toxicity and removal from wastewaters by means of ozonation. Water Research. 2006;40: 630–638. doi:10.1016/j.watres.2005.11.023

3. Sheikhlangi Z, Gharaei A, Harijani JM, Davari SA, Hassanein P, Rahdari A. Toxicological effects of meloxicam on physiological and antioxidant status of common carp (Cyprinus carpio). Veterinary Medicine and Science. 2023;9: 2085–2094. doi:10.1002/vms3.1207

4. Zhang S-Q, Li P, Zhao X-L, He S-W, Xing S-Y, Cao Z-H, et al. Hepatotoxicity in carp (*Cyprinus carpio*) exposed to environmental levels of norfloxacin (NOR): Some latest evidences from transcriptomics analysis, biochemical parameters and histopathological changes. Chemosphere. 2021;283: 131210. doi:10.1016/j.chemosphere.2021.131210

5. Diogo BS, Rodrigues S, Antunes SC. Mixture Matters: Exploring the Overlooked Toxicity of Sulfamethoxazole and Trimethoprim in Aquatic Environments. Environmental Toxicology. 2025;40: 1277–1293. doi:10.1002/tox.24528

6. Sanchez-Aceves LM, Gómez-Olivan LM, Pérez-Alvarez I, Orozco-Hernández JM, Gutiérrez-Noya VM, Rosales-Pérez KE, et al. Multilevel neurotoxicity of venlafaxine in zebrafish: Behavioral impairment, biomolecular dysregulation, and brain tissue damage. Environmental Research. 2025;285: 122328. doi:10.1016/j.envres.2025.122328

7. Adedara IA, Gonçalves FL, Mohammed KA, Borba JV, Canzian J, Resmim CM, et al. Waterborne atenolol disrupts neurobehavioral and neurochemical responses in adult zebrafish. Environ Sci Pollut Res. 2024;31: 49200–49213. doi:10.1007/s11356-024-34428-x

8. Lee S, Jung D, Kho Y, Ji K, Kim P, Ahn B, et al. Ecotoxicological assessment of cimetidine and determination of its potential for endocrine disruption using three test organisms: *Daphnia magna*, *Moina macrocopa*, and *Danio rerio*. Chemosphere. 2015;135: 208–216. doi:10.1016/j.chemosphere.2015.04.033

9. Cardoso-Vera JD, Gómez-Oliván LM, Islas-Flores H, García-Medina S, Elizalde-Velázquez GA, Orozco-Hernández JM, et al. Multi-biomarker approach to evaluate the neurotoxic effects of environmentally relevant concentrations of phenytoin on adult zebrafish *Danio rerio*. Science of The Total Environment. 2022;834: 155359. doi:10.1016/j.scitotenv.2022.155359

10. Li Q, Zhou Y, Kang W, Tang X, Hu B, Tang Z, et al. Environmental Concentrations of Herbicide Prometryn Increase the Susceptibility of Reef-Building Corals to Nitrate Stress. Environ Sci Technol. 2026;60: 16552–16565. doi:10.1021/acs.est.6c06486

11. Aktar W, Sengupta D, Chowdhury A. Impact of pesticides use in agriculture: their benefits and hazards. Interdisciplinary Toxicology. 2009;2: 1. doi:10.2478/v10102-009-0001-7

12. Jan-Roblero J, Cruz-Maya JA. Ibuprofen: Toxicology and Biodegradation of an Emerging Contaminant. Molecules. 2023;28: 2097. doi:10.3390/molecules28052097

13. Park J, An G, Park H, Hong T, Lim W, Song G. Developmental defects induced by thiabendazole are mediated via apoptosis, oxidative stress and alteration in PI3K/Akt and MAPK pathways in zebrafish. Environment International. 2023;176: 107973. doi:10.1016/j.envint.2023.107973

14. Lin Y, Shi R, Wang M, Wang Y, Han Y, Ma Y, et al. MCPA-Na exposure in aquatic systems: disruption of pathways and increased susceptibility to infection in fish. Aquatic Toxicology. 2025;284: 107405. doi:10.1016/j.aquatox.2025.107405

15. Rodrigues S, Gonçalves C, Golovko O, Antunes SC. Ecotoxicological impacts of caffeine on Daphnia magna: insights from acute to chronic exposures. Ecotoxicology. 2025;34: 1798–1814. doi:10.1007/s10646-025-02955-z

16. Motúzová T, Gavlová A, Smutná K, Řepecká L, Vráblová M. Environmental Impact of DEET: Monitoring in Aquatic Ecosystems and Ecotoxicity Assessment. ACS EST Water. 2025;5: 6342–6352. doi:10.1021/acsestwater.5c00489

17. Bubna GA, Lima RB, Zanardo DYL, dos Santos WD, Ferrarese M de LL, Ferrarese-Filho O. Exogenous caffeic acid inhibits the growth and enhances the lignification of the roots of soybean (*Glycine max*). Journal of Plant Physiology. 2011;168: 1627–1633. doi:10.1016/j.jplph.2011.03.005

18. Gaja MA, Knapp JS. Removal of 2-mercaptobenzothiazole by activated sludge: a cautionary note. Water Research. 1998;32: 3786–3789. doi:10.1016/S0043-1354(98)00146-8

19. Zampelli S, Verna R. Environmental Pollution from Pharmaceuticals. Life. 2025;15: 1341. doi:10.3390/life15091341
