# Supplementary material for: Immobilized horseradish peroxidase hybrid biocatalysts for degradation of pharmaceuticals and other emerging micropollutants
Source: PLoS One. 2026 Sep 2;21(9):e0355285. doi: 10.1371/journal.pone.0355285 (PMC13537563; doi:10.1371/journal.pone.0355285)
Supplement: Supplementary file 3 [file pone.0355285.s010.docx]

S3 Table: MRM parameters for all 21 EPs.

| **No.** | **Emerging Pollutant** | **Retention time (min)** | **Precursor Ion (*m/z*)** | **Product Ion (*m/z*)** | **Polarity** | **Collision Energy (V)** |
| --- | --- | --- | --- | --- | --- | --- |
| 1 | Roxithromycin | 11.6 | 837 | 680 | Positive | 20 |
| 2 | Lincomycin-HCl | 7.6 | 407 | 359 | Positive | 20 |
| 3 | Meloxicam | 12.8 | 352 | 115 | Positive | 6 |
| 4 | Norfloxacin | 8.2 | 320 | 302 | Positive | 20 |
| 5 | Trimethoprim | 7.9 | 291 | 230 | Positive | 20 |
| 6 | Venlafaxine-HCl | 9.4 | 278 | 260 | Positive | 10 |
| 7 | Atenolol | 7.1 | 267 | 190 | Positive | 20 |
| 8 | SMX | 9.3 | 254 | 156 | Positive | 20 |
| 9 | Cimetidine | 6.9 | 253 | 159 | Positive | 10 |
| 10 | Phenytoin | 11.1 | 253 | 182 | Positive | 10 |
| 11 | Prometryn | 11.6 | 242 | 158 | Positive | 30 |
| 12 | Fluometuron | 11.7 | 233 | 72 | Positive | 30 |
| 13 | Ibuprofen | 14.4 | 207 | 161 | Positive | 20 |
| 14 | Thiabendazole | 7.6 | 202 | 175 | Positive | 30 |
| 15 | MCPA | 12 | 201 | 125 | Positive | 13 |
| 16 | Caffeine | 7.8 | 195 | 138 | Positive | 30 |
| 17 | DEET | 11.9 | 192 | 119 | Positive | 30 |
| 18 | Caffeic acid | 7.8 | 181 | 163 | Positive | 20 |
| 19 | MBT | 10.6 | 168 | 135 | Positive | 30 |
| 20 | Furosemide | 11 | 329 | 285 | Negative | 15 |
| 21 | Hydrochlorothiazide | 6.4 | 296 | 269 | Negative | 20 |
