# Supplementary material for: Immobilized horseradish peroxidase hybrid biocatalysts for degradation of pharmaceuticals and other emerging micropollutants
Source: PLoS One. 2026 Sep 2;21(9):e0355285. doi: 10.1371/journal.pone.0355285 (PMC13537563; doi:10.1371/journal.pone.0355285)
Supplement: Supplementary file 4 [file pone.0355285.s011.docx]

S4 Table: Secondary structure correlation analysis

| Pearson Correlations | Jaccard vs % Degradation | | Fe_distance vs % Degradation | | Catalytic_core_contacts vs % Degradation | |
| --- | --- | --- | --- | --- | --- | --- |
|  | r | p | r | p | r | p |
| HRP only, free | -0.181 | 0.503 | -0.142 | 0.599 | 0.212 | 0.431 |
| HRP+HOBT, free | 0.012 | 0.965 | -0.162 | 0.548 | 0.301 | 0.257 |
| HRP only, occluded via TiO_2_ | 0.159 | 0.662 | 0.04 | 0.912 | -0.148 | 0.683 |
| HRP+HOBT, occluded via TiO_2_ | -0.063 | 0.863 | 0.437 | 0.207 | -0.126 | 0.73 |
| HRP only, occluded via ZnO | 0.209 | 0.563 | 0.001 | 0.999 | 0.325 | 0.359 |
| HRP+HOBT, occluded via ZnO | 0.121 | 0.74 | -0.278 | 0.436 | -0.124 | 0.734 |
