# Supplementary material for: Immobilized horseradish peroxidase hybrid biocatalysts for degradation of pharmaceuticals and other emerging micropollutants
Source: PLoS One. 2026 Sep 2;21(9):e0355285. doi: 10.1371/journal.pone.0355285 (PMC13537563; doi:10.1371/journal.pone.0355285)
Supplement: Supplementary file 5 [file pone.0355285.s012.docx]

S5 Table: Secondary structure group comparison

| Structure_type | High_n | High_mean  _pct | High_std  _pct | Low_n | Low_mean  _pct | Low_std_  pct | U_  statistic | p_  value |
| --- | --- | --- | --- | --- | --- | --- | --- | --- |
| helix_pct | 3 | 22.2 | 6.938 | 13 | 22.838 | 7.179 | 20 | 1 |
| beta_pct | 3 | 0 | 0 | 13 | 0 | 0 | 19.5 | 1 |
| coil_pct | 3 | 19.767 | 8.164 | 13 | 26.731 | 4.42 | 9 | 0.1708 |
| turn_pct | 3 | 58.033 | 4.081 | 13 | 50.423 | 6.26 | 33 | 0.0741 |
