# Supplementary material for: Immobilized horseradish peroxidase hybrid biocatalysts for degradation of pharmaceuticals and other emerging micropollutants
Source: PLoS One. 2026 Sep 2;21(9):e0355285. doi: 10.1371/journal.pone.0355285 (PMC13537563; doi:10.1371/journal.pone.0355285)
Supplement: Supplementary file 6 [file pone.0355285.s013.docx]

S6 Table: Per-ligand secondary structure comparison in free and occluded HRP
